# Genome-wide identification of notochord enhancers comprising the regulatory landscape of the brachyury locus in mouse

**DOI:** 10.1242/dev.202111

**Published:** 2023-11-09

**Authors:** Dennis Schifferl, Manuela Scholze-Wittler, Alba Villaronga Luque, Milena Pustet, Lars Wittler, Jesse V. Veenvliet, Frederic Koch, Bernhard G. Herrmann

**Affiliations:** Max Planck Institute for Molecular Genetics, Department Developmental Genetics, Ihnestr. 63-73, 14195 Berlin, Germany

**Keywords:** Mouse, Embryo, Development, Notochord, Brachyury, Enhancer

## Abstract

The node and notochord are important signaling centers organizing the dorso-ventral patterning of cells arising from neuro-mesodermal progenitors forming the embryonic body anlage. Owing to the scarcity of notochord progenitors and notochord cells, a comprehensive identification of regulatory elements driving notochord-specific gene expression has been lacking. Here, we have used ATAC-seq analysis of FACS-purified notochord cells from Theiler stage 12-13 mouse embryos to identify 8921 putative notochord enhancers. In addition, we established a new model for generating notochord-like cells in culture, and found 3728 of these enhancers occupied by the essential notochord control factors brachyury (T) and/or Foxa2. We describe the regulatory landscape of the *T* locus, comprising ten putative enhancers occupied by these factors, and confirmed the regulatory activity of three of these elements. Moreover, we characterized seven new elements by knockout analysis in embryos and identified one new notochord enhancer, termed *TNE2*. *TNE2* cooperates with *TNE* in the trunk notochord, and is essential for notochord differentiation in the tail. Our data reveal an essential role of Foxa2 in directing T-expressing cells towards the notochord lineage.

## Teaser

**Summary:** Multi-omics assays of purified embryonic and *in vitro*-generated cells identify thousands of notochord enhancers including TNE2, which is essential for notochord function and tail development of the mouse embryo.

## INTRODUCTION

The notochord is a rod-like structure situated below the neural tube and extending all along the trunk and tail of the embryo. As the defining characteristic of chordates, it serves two major functions: to provide structural stability to the developing embryo and to secrete signals for patterning of neighboring tissues (Stemple, 2005). It comprises an epithelium wrapped around a core of vacuolated cells, which makes the structure both solid and laterally flexible. In aquatic animals, these characteristics are essential and allow the embryo to swim. In fact, in some primitive fish the notochord serves as the main axial skeleton. In most chordates and all mammals, however, the notochord is a transient embryonic signaling center, which regresses during fetal development and forms the nucleus pulposus integrated in the intervertebral discs (Choi et al., 2008; McCann et al., 2012).

Adjoining organ anlagen of all three germ layers, the notochord lies ventral to the neuroectoderm, dorsal to the gut endoderm, and is flanked by somitic mesoderm. Patterning of the neural tube via secretion of Hedgehog ligands and Bmp antagonists from the notochord is a well-described signaling function (Briscoe and Ericson, 1999; McMahon et al., 1998). Further, the node induces the left-right axis and the notochord provides signals for patterning the endoderm during organogenesis (Cleaver and Krieg, 2001; Shiratori and Hamada, 2006). In the mesodermal lineage, the notochord is required for somite patterning (Brand-Saberi et al., 1993; Fan and Tessier-Lavigne, 1994), sclerotome induction, vertebral column differentiation and segmentation (Ward et al., 2018).

Axial mesoderm emerges during gastrulation and forms the prechordal plate, the anterior head process, and the node, which gives rise to trunk and tail notochord (Sulik et al., 1994; Tam and Beddington, 1987; Wymeersch et al., 2019; Yamanaka et al., 2007). Morphogenesis of these structures is controlled by a set of developmental transcription factors, in particular Foxa2 and brachyury (T; also known as Tbxt). The endodermal master regulator Foxa2 is essential for notochord specification at all axial levels (Ang and Rossant, 1994; Weinstein et al., 1994). Node formation and specification of trunk and tail notochord is controlled by the pan-mesodermal regulator brachyury in a dosage-dependent manner (Herrmann, 1995; Herrmann et al., 1990; Stott et al., 1993). In addition, the homeobox transcription factor Noto is required for tail notochord and disrupted in the truncate mutant, which displays a shortened tail phenotype (Abdelkhalek et al., 2004; Plouhinec et al., 2004; Zizic Mitrecic et al., 2010). During trunk notochord morphogenesis, Noto functions synergistically with Foxa2, before it becomes essential for tail notochord maintenance (Yamanaka et al., 2007). Of the three transcription factors, Noto is the only factor that is exclusively expressed in axial mesoderm precursors from embryonic day (E) 7.5.

The combined activities of T, Foxa2, Noto and other transcription factors establish the gene regulatory network that governs notochord morphogenesis (Di Gregorio, 2020; Matsumoto et al., 2007; Passamaneck et al., 2009; Song et al., 2023; Yamanaka et al., 2007). So far, few notochord enhancers have been identified in mice, including *TNE*, *Foxa2 NE*, *NOCE* and *Sfpe2* at the *T*, *Foxa2*, *Noto* and *Shh* loci respectively (Alten et al., 2012; Jeong and Epstein, 2003; Nishizaki et al., 2001; Schifferl et al., 2021). We hypothesized that, in addition to *TNE*, which is active during trunk notochord specification and essential for tail development, a second enhancer compensating for its loss must be located upstream of the *T* gene (Schifferl et al., 2021). Enhancers can be predicted by assessing chromatin accessibility, indicative histone modifications and transcription factor binding (Heintzman et al., 2007; Rada-Iglesias et al., 2011; Visel et al., 2009). Previous studies on notochord enhancers were limited owing to the small number of cells available from embryonic material and the restricted accessibility of axial mesoderm (Tamplin et al., 2011).

In this study, we present a comprehensive and integrated approach utilizing assay for transposase-accessible chromatin with sequencing (ATAC-seq), chromatin immunoprecipitation with sequencing (ChIP-seq) and transcriptome profiling to identify notochord enhancers throughout the genome and their corresponding target genes. We elucidate the cis-regulatory landscape of the *T* locus comprising multiple enhancers, and identify crucial enhancers required for *T* expression in the notochord and essential for notochord formation and differentiation.

## RESULTS AND DISCUSSION

### Genome-wide identification of notochord-specific enhancers bound by T and/or Foxa2

**Fig. 1.**
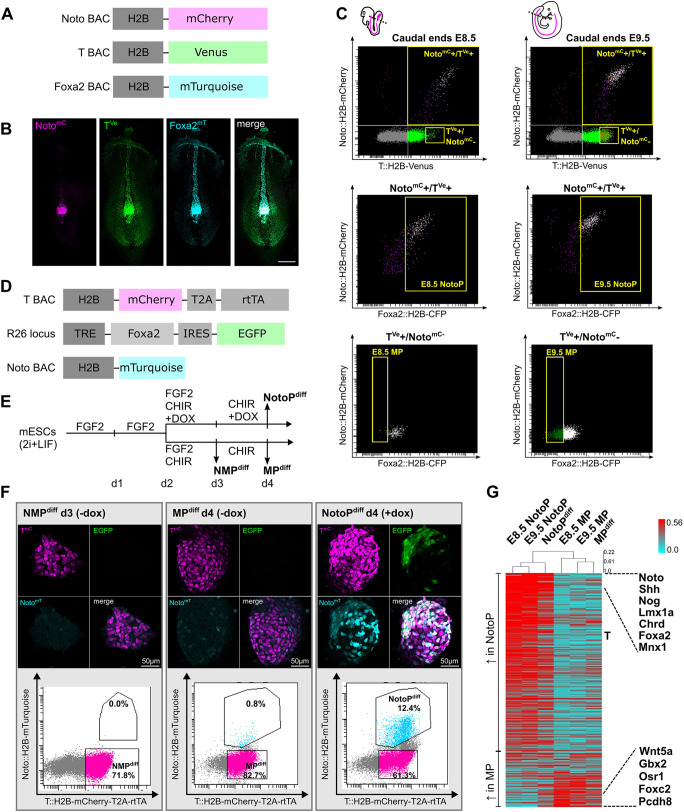
**Derivation of NotoP and MP progenitor cells *in vivo* and *in vitro*.** (A) BAC constructs integrated into an mESC line for the establishment of a Noto^mC^/T^Ve^/Foxa2^mT^ triple reporter line. (B) Maximum intensity projection of images acquired by confocal microscopy of an E8.5 Noto^mC^/T^Ve^/Foxa2^mT^ mouse embryo. Scale bar: 200 µm. (C) FACS gating for purification of NotoPs and MPs from caudal ends of E8.5 and E9.5 embryos dissected at the somite border (as indicated by dotted lines in schematics; pink lines indicate the notochord). (D) Genetic modifications of an mESC line used for T-driven, Dox-inducible overexpression of Foxa2. (E) Differentiation scheme for the *in vitro* generation of NMPs, MPs and NotoPs from mESCs carrying the modifications shown in D; modified from Gouti et al., 2014. (F) Images of differentiated colonies acquired by confocal microscopy, and corresponding FACS profiles. (G) Ranked heat map showing per gene normalized FPKM values of 418 differentially expressed genes (3-fold change in NotoPs versus MPs at both E8.5 and E9.5) and corresponding *in vitro*-generated cells (NotoP^diff^; MP^diff^). Genes are sorted by average fold change in E8.5 and E9.5 samples. Examples of notochord (top) and mesoderm (bottom) progenitor marker genes are indicated on the right.

The notochord is characterized by co-expression of brachyury and Foxa2, which are also expressed in neuro-mesodermal progenitors (NMPs) and mesoderm or endoderm, respectively. Noto, by contrast, is notochord specific. To characterize the enhancer landscape of the notochord, we engineered a Noto::H2B-mCherry/T::Venus/Foxa2::mTurquoise triple reporter mouse embryonic stem cell (mESC) line allowing the isolation of putative notochord (Noto^+^/T^+^/Foxa2^+^) progenitor cells (NotoPs; Fig. 1A). The reporter line was used for the generation of embryos by diploid morula aggregation (Fig. 1B). The caudal ends of E8.5 and E9.5 embryos were dissected at the somite border, and Noto^mC+^/T^Ve+^/Foxa2^mT+^ cells were purified by fluorescence-activated cell sorting (FACS) (Fig. 1C). For comparison, we also purified trunk notochord (Fig. S1A) and paraxial mesoderm progenitors (MPs; T^Ve+^/Noto^mC−^/Foxa2^mT−^; Fig. 1C). E8.5- and E9.5-derived cells were used for transcriptome profiling (Fig. 1C, Fig. S1A), cell pools from Theiler stage 12 and 13 embryos for ATAC-seq analysis (Fig. S1B-D).

Embryonic notochords do not provide sufficient cell numbers for generating ChIP-seq data from FACS-sorted material. To overcome this limitation, we developed an *in vitro* model for differentiation of mESCs into NotoPs. In parallel, we produced MPs under similar experimental conditions. Notochord cells require high levels of T in combination with Foxa2. Therefore, we generated an mESC line allowing doxycycline (Dox)-inducible expression of Foxa2 and eGFP, driven by reverse tetracycline-controlled transactivator (rtTA; Gossen et al., 1995) expressed under control of the T promoter and enhancers. In addition, this line carries a Noto^mT^ reporter (Fig. 1D). We followed a well-established protocol for generating NMPs on day 3 of culture (Fig. 1E; Gouti et al., 2014). CHIR 99021 (CHIR) treatment for one more day generated MPs (herein termed MP^diff^ to distinguish them from embryonic MPs), as previously shown. However, parallel treatment with CHIR and Dox for 2 days (d2-d4) caused Foxa2 and eGFP expression, and efficiently generated Noto^mT+^ cells, as shown by FACS and fluorescent microscopy (Fig. 1F; Fig. S2A). To investigate whether these cells resemble embryonic NotoPs, we isolated Noto^mT+^/T^mC+^ (NotoP^diff^) cells (Fig. 1F) and performed RNA sequencing (RNA-seq). In parallel, we analyzed MP^diff^ cells (T^mC+^/Noto^mT−^). We compared the transcriptomes of *in vitro*-generated cells with embryonic cells isolated from E8.5 and E9.5 embryos. For embryonic NotoP and MP cells, we identified 319 and 109 specifically expressed genes, respectively [3-fold change of fragments per kilobase of transcript per million mapped reads (FPKM) value in NotoP versus MP at E8.5 and E9.5], including many known markers for both cell types (Fig. 1G, Fig. S2B, Table S1). The comparison revealed striking similarity between the expression profiles of embryonic and *in vitro*-generated cells. Considerable overlap was observed between notochord gene sets identified in NotoP cells and those reported in earlier studies (Fig. S2C, Table S2) (Tamplin et al., 2011; Wymeersch et al., 2019; Peck et al., 2017). Immunofluorescent staining (Fig. S2D) and qPCR confirmed that T and Foxa2 are upregulated in NotoP^diff^ cells, whereas expression of the paraxial mesoderm marker *Tbx6* is low (for detailed qPCR data analysis, see Fig. S2E). We conclude that our *in vitro* model is suitable for efficiently generating notochord-like cells strongly resembling embryonic NotoPs.

**Fig. 2.**
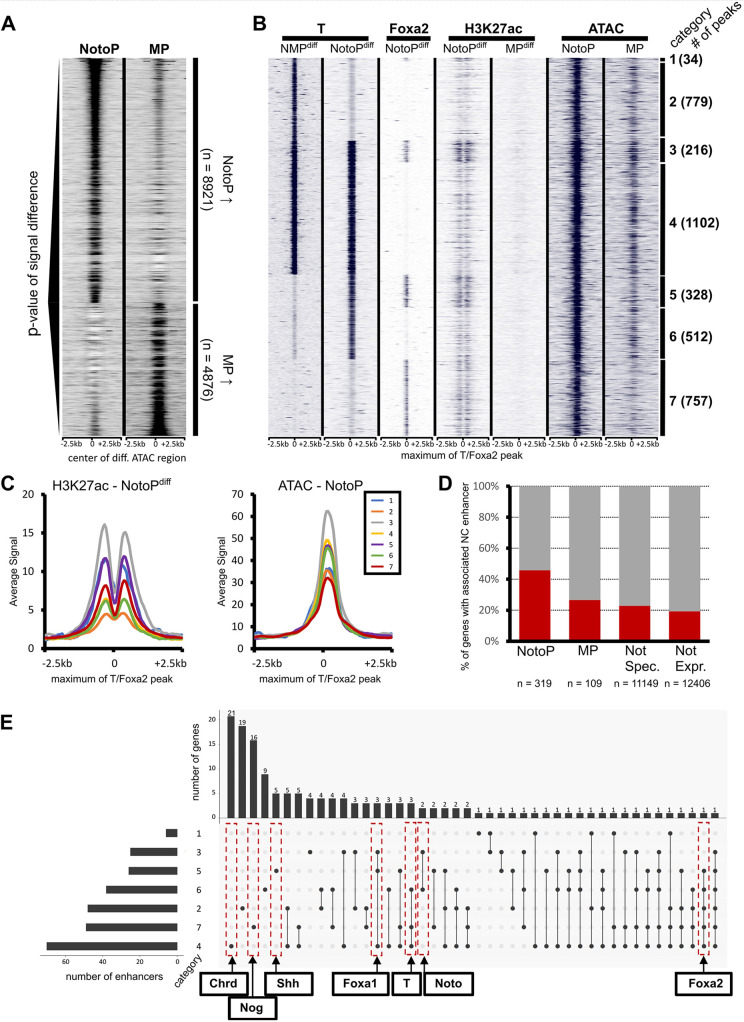
**Genome-wide prediction and analysis of notochord enhancers.** (A) Heatmap showing ATAC-seq signals of NotoP and MP cells around 8921 notochord enhancer candidates defined by differential peak detection, sorted by *P*-values. (B) Heatmap of 3728 differential NotoP accessible sites bound by T and/or Foxa2 in NotoP^diff^ cells. Putative enhancers are categorized into all possible combinations of T and Foxa2 binding combinations (1-7, right) and the number of putative enhancers per category is shown in parentheses. (C) Average profiles of H3K27ac and ATAC-seq signals for all enhancer categories. (D) Bar plot quantifying the association of putative notochord enhancers to NotoP, MP, not specifically expressed (Not Spec.) and not expressed (Not Expr.) genes. (E) UpSet plot for the 146 NotoP-specific genes showing their associated enhancers according to categories 1-7.

Next, we identified putative notochord enhancers. We performed ATAC-seq on embryonic NotoPs and MPs (Fig. S1B-D). Differential peak detection identified 8921 open regions outside of promoters (±5 kb from the transcription start site) with higher accessibility in NotoPs compared with 4876 regions with higher accessibility in MP cells (Fig. 2A; Fig. S3A). To characterize these accessible regions with respect to T and/or Foxa2 binding, we generated cultures enriched for NotoP^diff^ cells (CHIR^+^; Dox^+^; d4). We performed ChIP-seq for T and Foxa2 on bulk cultures. In addition, for determining specific chromatin signatures we FACS-purified NotoP^diff^ [CHIR^+^; Dox^+^; d4; cell population (P)7] and MP^diff^ (CHIR^+^; Dox^−^; d4; P5) cells as above (Fig. S2E) and analyzed several histone marks (H3K27ac, H3K4me3, H3K4me1 and H3K9me2) by ChIP-seq. For comparison, we used T ChIP-seq data previously generated from *in vitro-*differentiated NMPs (Koch et al., 2017).

The co-expression of T and Foxa2 in our *in vitro* model strikingly changed the chromatin landscape from a mesoderm signature (MP^diff^: CHIR^+^; Dox^−^; d4) to a notochord signature (NotoP^diff^: CHIR^+^; Dox^+^; d4) (Fig. 2B). Among the 8921 open regions differentially accessible in NotoPs versus MPs, we identified 3728 sites bound by T and/or Foxa2 in NotoP^diff^ cells (Fig. 2B). Using H3K27ac data as a strong indicator of enhancer activity (Creyghton et al., 2010), we found the highest signal in enhancers binding both T and Foxa2 [category (Cat) 3, followed by 5 and 1; Fig. 2C; Fig. S3B]. Cat 3 enhancers also showed the highest ATAC signal in embryonic NotoPs, and were also bound by T in NMPs. Cat 5 enhancers specifically occupied by T and Foxa2 in NotoP^diff^ cells showed somewhat lower ATAC and H3K27ac signal. These data suggest that Foxa2 and T might have additive effects on enhancer activation. Based on the H3K27ac signal, Foxa2 binding alone (Cat 7) seems to be more effective than T alone (Cat 2, 4, 6), whereas the accessibility of Cat 4, 5 and 6 enhancers appears to be equal. However, some of these differences may be caused by co-binding of additional factors not analyzed in this study.

A considerable fraction (2393/3728; 64.2%) of notochord enhancers showed T binding alone (Cat 2, 4 and 6), and a smaller fraction (757/3728; 20.3%) only Foxa2 binding (Cat 7). Most of the T-binding enhancers (2131/3728; 57.1%) were also T bound in NMPs, but 840 (22.5%) T-bound enhancers were only occupied in notochord cells. Thus, Foxa2 co-expression with T opens chromatin at enhancers not occupied by T when Foxa2 is not expressed, and this cooperative action changes the genomic landscape to a notochord signature.

Next, we determined the enhancer presence in the vicinity of 319 genes specifically upregulated in NotoP cells (Fig. 1G). We found at least one notochord enhancer each in the gene bodies or genomic intervals comprising the intergenic regions and gene bodies of the immediate neighbors of 146 (46.4%) of these genes (Fig. 2D). Fig. 2E shows the number of genes in relation to the category(s) and number of enhancers found in their neighborhood, with examples of prominent notochord marker genes. Strikingly, most of the latter are associated with several putative enhancers falling into different enhancer categories and none of them is associated with a T-NMP^diff^ peak (Cat 2) alone. Genome browser screenshots of important markers are given in Fig. S4, and of housekeeping genes for control in Fig. S5. In addition to the notochord enhancers described previously, we found new putative enhancers, for example for *Noto*, *Foxa2*, *Chrd*, *Nog* and *Foxa1* (Schifferl et al., 2021; Alten et al., 2012; Nishizaki et al., 2001; Jeong and Epstein, 2003). A list of all notochord enhancer candidates identified here and their associated genes, as well as the list of the 319 NotoP genes and the categories of their associated enhancers, is given in Tables S3 and S4, respectively. Our data provide an important resource of putative notochord enhancers.

### The regulatory landscape of the *T* locus in notochord and mesoderm

**Fig. 3.**
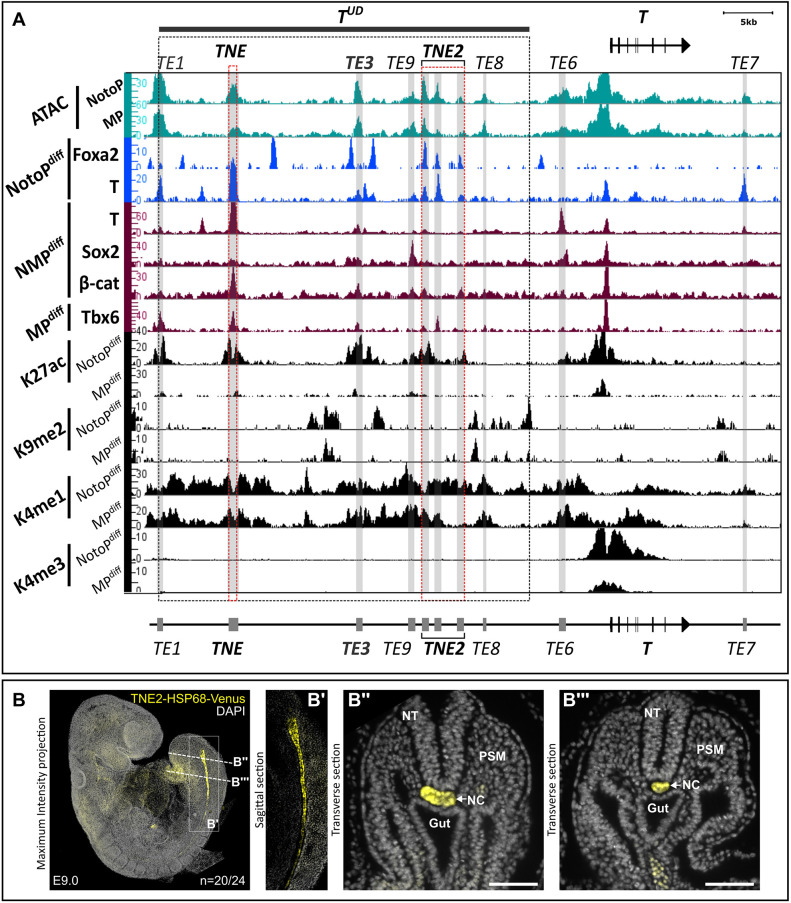
**Identification of putative enhancers at the mouse *T* locus.** (A) Genome browser snapshot showing the mouse *T* locus with signal density tracks of ATAC-seq, transcription factor ChIP-seq and histone 3 ChIP-seq data. Track maxima are normalized to the number of mapped reads for each antibody. Enhancer elements are indicated as gray boxes and shown schematically below the ChIP-seq tracks; TNE and TNE2 are highlighted by red dashed lines. The *T^UD^* and *TNE* regions were described previously (Schifferl et al., 2021). (B) *TNE2*-driven Venus reporter expression in the notochord of an E9.0 embryo. Nuclei are stained with DAPI (gray). Left: maximum intensity projections of confocal microscopy. (B′) Sagittal optical midline section. (B″,B‴) Transverse optical sections acquired by light-sheet microscopy at the axial levels indicated in B. NC, notochord; NT, neural tube; PSM, presomitic mesoderm. Scale bars: 100 µm.

Next, we used our integrated ATAC and ChIP data to characterize further the regulatory landscape of the *T* locus in notochord and mesoderm. We previously identified the *TNE* enhancer, which is essential for tail notochord development; however, this enhancer alone does not fully explain the severe loss of notochord and tailbud phenotype of a 37 kb deletion (*T^UD^*), suggesting that additional notochord and mesoderm elements are present in this region (Schifferl et al., 2021). Our ATAC-seq data of the *T* locus showed ten peaks, including *TNE* (Fig. 3).

Three ATAC-seq peaks in close vicinity to each other were bound by T and Foxa2 and showed enrichment for the active enhancer mark H3K27ac in NotoP^diff^ cells (Fig. 3). Therefore, we used a 4.4 kb fragment encompassing these T- and Foxa2-binding sites to perform an enhancer assay. The embryonic activity assay at E9.0 showed Venus reporter expression in the posterior notochord, decreasing towards the head, identifying the 4.4 kb fragment as a notochord-specific enhancer (Fig. 3B-B‴). We designated this enhancer *T locus notochord enhancer 2* (*TNE2*).

In addition, based on ATAC peaks and, in a subset, also H3K27 acetylation, we defined several more candidate *T* locus enhancers, *TE1*, *TE3*, *TE9* and *TE8*, within the *T^UD^* region (Schifferl et al., 2021), as well as *TE6* −5 kb upstream and *TE*7 13 kb downstream of the *T* transcription start site (Fig. 3). We integrated ChIP-seq data for T, Sox2, β-catenin and Tbx6 published previously, to relate these enhancers to regulatory processes occurring in NMPs and during mesoderm formation (Koch et al., 2017). For example, Sox2 acting antagonistically to T in the neural versus mesodermal lineage choice, binds to *TE6* and *TE9* in NMPs, whereas the WNT signal mediator β-catenin, which cooperates with T in NMPs and NotoPs, binds to *TE3* and *TNE*. In contrast, Tbx6, which has a repressive effect on NMP maintenance and *T* expression, is detected at *TE1*, *TNE*, *TNE2* and possibly *TE3*. All four TFs also bind at the *T* promoter.

We assayed the activity of *TE3*, characterized by T peaks flanked by Foxa2 binding in NotoP^diff^ cells, using the HSP68-Venus reporter. This element showed expression in the tailbud mesoderm, posterior neural tube and gut of the tail at E10.5, but not in the notochord (Fig. S6A-A‴). These data suggest that *TE3* acts as enhancer in tailbud NMPs. The significance of the flanking Foxa2 peaks is unclear.

Moreover, we assayed the putative enhancer *TE7*, which is located downstream of the *T* gene and was not identified by chromatin marks or differential ATAC analysis, but showed a small ATAC peak and T binding in NotoP^diff^ cells. β-Galactosidase reporter activity was detected in the entire notochord of E9.0 embryos, albeit with increasing staining towards the head (Fig. S6B). Thus, *TE7* identifies another notochord enhancer. The data suggest that the activity of *TE7* is increasing during notochord differentiation, which might explain why it was not detected in our differential ATAC-seq data derived from caudal end NotoPs.

### A new *T* locus notochord enhancer, *TNE2*, cooperates with *TNE* in notochord development

Next, we investigated the function of several putative *T* locus enhancers by employing the CRISPR/Cas9 system to generate a series of knockout (KO) mESC lines and embryos carrying the *Noto::H2B-mCherry* (Noto^mC^) reporter (Fig. S7, Table S5). We generated mutant embryos by tetraploid complementation assays and evaluated the phenotype between E9.5 and E12.5 by visualizing the Noto^mC^ reporter as well as T and Sox2 protein expression. Deletions of *TE1*, *TE3*, *TE9* and *TE*7 did not result in embryonic defects enhancing the phenotypes of the corresponding parental line, which contained either the wild-type *T* locus or the heterozygous *T^LD^* deletion covering the entire *T* locus (Schifferl et al., 2021). The data suggest that these cis control elements either have no critical function or are redundant (Table S6).

**Fig. 4.**
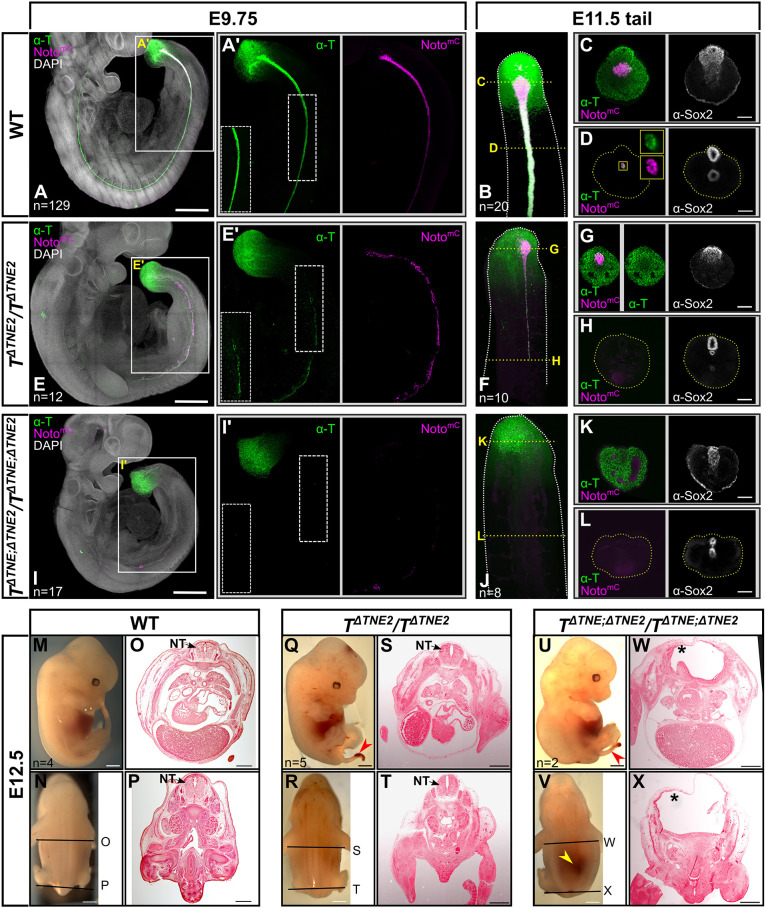
***TNE2* is an essential notochord enhancer of brachyury and interacts with *TNE*.** (A,A′,E,E′,I,I′) Maximum intensity projections of E9.75 embryos with Noto^mC^ reporter signal, immunostaining for T (green) and DAPI nuclear staining (gray). Scale bars: 500 µm. Boxed areas are magnified in single channels. To visualize the lower T signal of the mutant notochord in (E′), maximum brightness was equally adjusted in the insets at the bottom left of A′,E′,I′. (B-D,F-H,J-L) Maximum intensity projections of E11.5 tails with immunostaining for T (green) and the Noto^mC^ reporter signal (magenta) are shown in B,F,J. Yellow dashed lines indicate the position of optical sections shown in C,D,G,H,K,L, Immunostaining for Sox2 is shown in white, insets in D show a magnification of the notochord (boxed). Dotted lines indicate the circumference of the section. Scale bars: 100 µm. (M,N,Q,R,U,V) Lateral and dorsal views of E12.5 embryos. Red or yellow arrowheads indicate the tail phenotype or neural tube defect, respectively. Scale bar: 1 mm. (O,P,S,T,W,X) Histological sections at the axial levels indicated in N,R,V. Asterisks indicate lack of a neural tube (NT) (W,X). Scale bars: 500 µm. WT, wild type.

However, homozygous deletion of *TNE2* resulted in a notochord deficiency phenotype. In E9.75 *T^ΔTNE2/ΔTNE2^* mutant embryos, less T protein was detected in the trunk notochord marked by Noto^mC^ than in wild-type embryos, and the notochord was partially disrupted at the hindlimb level (Fig. 4A,A′,E,E′). At E11.5, the Noto^mC+^ notochord progenitor domain was present in the outgrowing tailbud, but the number of Noto^mC+^ cells in the midline was reducing quickly towards the anterior and the notochord was not formed (Fig. 4B-D,F-H). The reduced T protein level in the trunk notochord was apparently still sufficient for trunk development, but tail notochord formation was not supported resulting in a tail-less phenotype at E12.5 (Fig. 4M-T).

In E9.75 *T*^Δ*TNE;* Δ*TNE2/*Δ*TNE;* Δ*TNE2*^ double KO embryos, T expression was detected in the tailbud, but not in notochord progenitors, and the notochord was not formed (Fig. 4I,I′). A few cells with weak Noto^mC+^ signal were visible in gut endoderm, indicating improper specification (Fig. 4I′). Neither Noto^mC+^ cells nor a notochord were detected in the tailbud at E11.5 (Fig. 4J-L). Immunofluorescence staining for Olig2 and Nkx2-2 revealed neural tube patterning defects (Fig. S8). Olig2 expression was shifted ventrally, whereas Nkx2-2 was not detected. Consistent with the latter, neural tube differentiation was severely affected at E12.5, resulting in embryonic lethality (Fig. 4U-X).

**Fig. 5.**
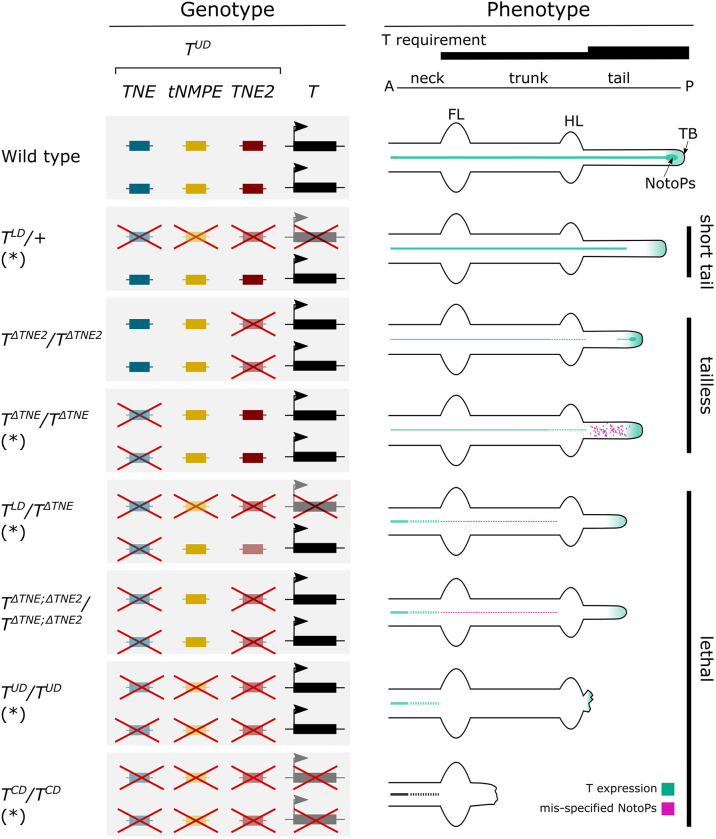
**Schematic view of *T* enhancer function in axial development.** Axial elongation depends on *T* expression in the tailbud and in the notochord (green line). Notochord formation in the trunk requires at least one of the enhancers TNE or TNE2, tail notochord formation both. *T* enhancers located in the *T^UD^* deletion and the *T* gene are shown as boxes on the left; loss-of-function mutations are indicated by red crosses. A, anterior; FL, forelimb bud; HL, hindlimb bud; P, posterior; TB, tailbud; tNMPE, tail NMP enhancer, defined by failure of tailbud outgrowth in *T^UD^*/*T^UD^* embryos. Dotted lines/areas indicate interrupted notochord (green) or mis-specified NotoPs (red), respectively. For details, see the text; data from Schifferl et al. (2021) are marked by an asterisk.

Our data show that *TNE* can compensate for the loss of *TNE2* during trunk notochord formation and vice versa (Fig. 5; Schifferl et al., 2021). The double KO phenotype shows that the combined activity of these two enhancers is required for notochord formation and differentiation in the trunk and tail. *TE7* is not able to compensate for the loss of *TNE* and *TNE2*. The reduced T protein level in either KO suggests that the two enhancers cooperate during trunk notochord development. The partial disruption of the notochord in the posterior trunk of either single KO suggests an increasing requirement for T expression in notochord progenitors along the axis, as has been reported previously (Stott et al., 1993; Herrmann, 1995). Alternatively, higher T activity may be required during progenitor formation in the node in order to generate sufficient cell numbers supporting notochord development throughout the trunk. Tail notochord formation, however, needs the combined activity of both enhancers.

The single loss-of-function phenotypes reveal different roles of *TNE* and *TNE2* in the tail. There, *TNE* is mainly required for notochord progenitor maintenance, notochord cell specification and presumably differentiation, given that embryos lacking *TNE2* still form Noto^mC+^ notochord progenitor cells, whereas in *TNE* mutants notochord cell specification is lost (Schifferl et al., 2021). *TNE2*, by contrast, is required for proper notochord cell differentiation, as in embryos lacking *TNE2* Noto^mC+^ cells are still present in the progenitor domain, but are quickly reducing in number in the midline toward the anterior and the notochord is lacking. Of note, our data suggest cooperative binding of T and Foxa2 at *TNE2*, whereas *TNE* appears to bind T and β-catenin, consistent with the role of canonical Wnt signaling in notochord specification (Ukita et al., 2009). Thus, T appears to act earlier in the lineage through *TNE*, followed by cooperative action with Foxa2 through *TNE2*. Our enhancer reporter assays suggest that *TE7* is active even later, upon further differentiation and possibly in the maintenance of the notochord, as during axis elongation the reporter activity is low in the posterior end and increasing towards the head. The strong *T^UD^* homozygous deletion phenotype reported previously is, at least with respect to the lack of the notochord, explained by the loss of *TNE* and *TNE2*, but *TE7* is not involved (Schifferl et al., 2021). However, the missing tailbud in the deletion mutant is not explained, as *TNE/TNE2* double KO embryos still form the tailbud and show some tail outgrowth. *TE3* driving expression in tailbud NMPs might be responsible for tail outgrowth in the absence of *TNE/TNE2*. However, our *TE3* deletion analysis did not show the expected loss of the tailbud in the absence of the enhancer, even in combination with the deletion of *TE9*, an enhancer candidate showing Sox2 and β-catenin binding in NMPs (Fig. S7; Table S6). Therefore, the control element(s) required for tailbud formation and tail outgrowth must remain undefined at this point.

The *in vitro* model for notochord progenitor formation introduced here is based on the essential role of both T and Foxa2 in node and notochord development. Accordingly, WNT (Chiron) induction is employed for parallel activation of *T* and *Foxa2*, the latter under control of the *T* promoter and enhancers comprised by a BAC construct. Because notochord is not derived from NMPs, our data suggest that *T* is already activated in epiblast stem cell-like cells. Strong upregulation of *Foxa2* supposedly directs a fraction of T^+^/Foxa2^+^ cells into the notochord lineage, whereas cells expressing no or low levels of Foxa2 become NMPs and take the mesodermal fate. However, whether NMPs and notochord-like cells have a common progenitor or derive from different subsets of epiblast stem cell-like cells remains unresolved at this point.

## MATERIALS AND METHODS

### General mESC procedures

All cell lines used in this study were derived from male mESCs of the G4 hybrid line 129S6/SvEvTac×C57BL/6Ncr (George et al., 2007). mESC clones were regularly tested for possible *Mycoplasma* contamination, using PCR Mycoplasma Test Kit II (Applichem, A8994) according to the manufacturer's recommendations.

### Generation and integration of BAC transgenes

For the generation of reporter and driver transgenes, BACs containing ∼200 kb of the C57/BL6 genome surrounding the mouse brachyury (RP24-530D23), *Foxa2* (RP23-254G2) and *Noto* (RP23-289M19) genes were obtained from BACPAC resources. The *T::H2B-Venus*, *T::H2B-mCherry-T2A-rtTA*, *Foxa2::H2B-mTurquoise* and *Noto::H2B-mTurquoise* BACS carrying neomycin, hygromycin, puromycin and blasticidin resistance cassettes, respectively, were engineered via Red/ET recombineering (Muyrers et al., 1999) as described previously (Schifferl et al., 2021). For random integration of BACs, 5 µg of each construct were linearized using PI-SceI (New England Biolabs, R0696S) and electroporated into 3×10^6^ mESCs. Approximately 30 h after electroporation, selection was initiated, as shown in Table S5. Selection medium was refreshed daily until single colonies were clearly visible after approximately 1 week. Single clones were picked and genotyped by PCR. Oligonucleotides used in this study are listed in Table S7.

### Generation of enhancer mutants

Homozygous *T*^Δ*TNE2*^*/T*^Δ*TNE2*^ single and *T*^Δ*TNE;* Δ*TNE2*^*/ T*^Δ*TNE;* Δ*TNE2*^ double mutants were generated from cells carrying the *Noto::H2B-mCherry* construct using CRISPR/Cas9 as reported previously (Schifferl et al., 2021).

### Recombinase-mediated cassette exchange

For the generation of enhancer reporter and Foxa2 overexpression cell lines, 3×10^5^ mESCs with a modified Rosa26 harboring locus (Vidigal et al., 2010) were co-transfected with 5 µg of linearized NE2-HSP68-Venus, TE3-HSP68-Venus, TE7-HSP68-bGal or TRE-Foxa2-IRES-EGFP constructs and 1 µg PGK-iCre vector using Lipofectamine 2000 (Invitrogen, 11668027). Cells were cultured in ES+LIF containing 350 µg/ml geneticin (Thermo Fisher Scientific, 10131027) for selection for successful recombination resulting in a switch from hygromycin to neomycin resistance.

### Generation of transgenic embryos

Transgenic mouse embryos were generated by diploid or tetraploid morula aggregation by the transgenic unit of the Max Planck Institute for Molecular Genetics in Berlin as described previously (Eakin and Hadjantonakis, 2006). All animal experiments were performed according to local animal welfare laws and approved by local authorities (covered by LaGeSo licenses G0243/18 and G0247/13).

### Embryo isolation

Timed pregnant foster mice were euthanized by carbon dioxide administration and cervical dislocation. For whole-mount immunofluorescence and tissue clearing, embryos were isolated from uteri in 4°C pre-cooled PBS, fixed using 4% paraformaldehyde (PFA) in PBS (Sigma-Aldrich, P6148) in 4 ml glass vials (Wheaton, 224882) and processed as described previously (Schifferl et al., 2021). For RNA-seq and ATAC-seq, embryos were isolated in M2 medium (Sigma-Aldrich, MR-015P). Samples were further dissected into the sub-regions of interest using forceps (Dumont, 11251-10). Tissue samples were kept on ice in M2 medium and processed subsequently.

### Whole-mount immunofluorescence and tissue clearing

Immunofluorescence staining and clearing procedures were performed on E9.75-E11.5 embryos as described previously (Schifferl et al., 2021). Antibodies are listed in Table S8.

### Whole-mount β-galactosidase staining

Embryos carrying the *TE7-HSP68-bGal* reporter were fixed for 30 min at 4°C and subsequently washed three times for 15 min in Rinse Buffer (50 mM EGTA, 0.1% deoxycholate, 0.2% NP-40, 20 mM MgCl_2_ in DPBS) at room temperature. After rinsing, embryos were incubated in staining solution [50 mM K_3_Fe(CN)_6_, 50 mM K_4_Fe(CN)_6_, 50 mM EGTA, 0.1% deoxycholate (100×), 0.2% NP-40 (100×), 0.2 M MgCl_2_, 1 mg/ml X-gal in DPBS] at 37°C overnight. Stained embryos were washed three times with PBS and stored in 4%PFA in PBS at 4°C for secondary fixation.

### FACS

For FACS of embryonic material, single-cell suspensions were prepared adding 100 µl Trypsin/EDTA to the sample. After incubation at room temperature for 5 min, trypsin was quenched by adding 200 µl PBS with 5% bovine serum albumin (BSA; Sigma-Aldrich, A8412).

For FACS of cell cultures, cells were washed twice in PBS and dissociated by trypsinization at 37°C for 10 min. Trypsin/EDTA was quenched using a double volume of 5% BSA in PBS, then cultures were resuspended and kept on ice until further procedure.

All samples were immediately filtered (35 µm mesh) and sorted on a FACS Aria II (Becton Dickinson) flow cytometer. For transcriptome analysis, cells were sorted into 350 µl RLT Plus buffer (QIAGEN, 1053393) containing 1% β-mercaptoethanol (Sigma-Aldrich, M6250) in 1.5 ml low-binding tubes (Thermo Fisher Scientific, 90410) and stored at −80°C until further procedure. For ChIP, cells were sorted into 5% BSA in PBS in BSA-coated glass tubes.

### Histology

PFA-fixed E12.5 embryos were dehydrated through an ethanol series (30%, 50%, 70%, 70%, 15 min each), processed in a MICROM STP 120 processor (Microm, 813150) and embedded in paraffin (Leica, 3801320) utilizing an EC 350-1 embedding station (Microm). Sections of 10 µm thickness were prepared using a rotary microtome (Microm, HM355S), transferred onto adhesion microscope slides (Menzel, K5800AMNZ72) and dried overnight at 37°C. Eosin (Merck, 109844) counterstaining was performed according to standard procedures and specimens were mounted in Enthellan (Sigma-Aldrich, 107960). Sections were imaged using an AxioZoom V16 stereomicroscope (Zeiss).

### Microscopy

Embryos were imaged using a Zeiss LSM880 laser-scanning microscope with Airyscan detector or Zeiss Light sheet LS Z1 with appropriate filters for DAPI, mTurquoise, EGFP, Venus, mCherry, Alexa 488 or Alexa 647. For light-sheet microscopy, specimens were cleared and embedded in 1.5% low melting agarose (Sigma-Aldrich, A9414) in PBS. Agarose columns containing the samples were cleared in RIMS overnight before acquisition. *z*-stacks of approximately 500 µm range were acquired with 8-10 µm intervals.

Processing was performed using ZEN Blue Version 3.1 and ZEN Black Version 2.3 (Zeiss) software. For the visualization of volumetric data, maximum intensity projections (Wallis et al., 1989) were generated using the maximum intensity projection volume rendering tool in ZEN Black. This tool generates 2D images from stacks of optical sections, displaying the brightest pixel (*xy*), along the *z*-axis in the according position (*xy*) on a 2D plane.

To visualize the weaker signal in mutant notochord cells (Fig. 4E′,I′), the maximum brightness parameter was adjusted, reducing the difference (maximum brightness−minimum brightness) by 50%.

### *In vitro* differentiation

NMP^diff^, MP^diff^ and NotoP^diff^ cells were derived from *T^mC−2A−irTA^/TRE::Foxa2/Noto^mT^* mESCs following an established protocol (Gouti et al., 2014) with previously described modifications (Koch et al., 2017). For NotoP^diff^ generation, 1 ng/ml Dox was applied from d2 to d4.

### RNA-seq

For transcriptome analysis of FACS-purified subpopulations, total RNA was isolated from 250 cells (or fewer; see Table S9) using the RNeasy MinElute kit (QIAGEN, 74204). RNA extraction was performed according to the manufacturer's protocol with an additional DNase digest step between two washes with 350 µl RW1. Therefore, reaction mixes of 10 µl DNase I (QIAGEN, 79254) and 1 µl (=10 U) DNase I (Roche, 4716728001) in 70 µl buffer RDD (QIAGEN, 1011132) were applied to the spin columns for a 15 min incubation at room temperature. Membranes were air dried for 10 min to remove the remains of ethanol and eluted in 15 µl H_2_O.

Sequencing libraries were prepared using the Ovation SoLo RNA-seq system (NuGEN) according to the manufacturer's recommendations, starting at step A.9 with 12 µl purified DNA. After each amplification step, libraries were quantified with the Qubit the High Sensibility DNA assay (Thermo Fisher Scientific, 12102). Library size was validated using DNA High Sensitivity Bioanalyzer chips (Agilent, 5067-4626).

cDNA library pools (150 nmol in 15 µl) with 16 bp barcode length (8 bp barcode+8 bp UMI) were sequenced on using the Illumina HiSeq4000 (for E8.5/E9.5 NotoP, MP and Noto trunk) or NextSeq 2000 (for Noto^diff^, MP^diff^, NMP^diff^) platforms.

Prior to mapping, the first five nucleotides of the forward and reverse reads were trimmed using fastx_trimmer (http://hannonlab.cshl.edu/fastx_toolkit/index.html) according to the manufacturer's instructions (NuGEN). The resulting reads were mapped to chromosomes 1-19, X, Y and M of the mouse mm10 genome using TopHat2 (v2.1.1) and bowtie (v1.2.2) (Kim et al., 2013; Langmead et al., 2009) and the RefSeq annotation in gtf format (UCSC), providing the options ‘--no-coverage-search --no-mixed --no-discordant -g1 --mate-inner-dist 250 --mate-std-dev 100 --library-type fr-secondstrand’. Read duplications resulting from the PCR amplification of the library were removed using the NuDup deduplication script provided by NuGEN (https://github.com/tecangenomics/nudup/). Wiggle files were generated with BEDTools version 2.23.0 (Quinlan and Hall, 2010), converted to bigwig format and visualized in the Integrated Genome Browser (Freese et al., 2016). FPKM values were calculated using Cufflinks version 2.2.1 (Trapnell et al., 2013) with options ‘-u --no-effective-length-correction -b’. Using FPKM values, per gene normalization, generation of heatmaps and non-hierarchical clustering of samples was performed in MeV (Howe et al., 2011).

### Gene set comparison

For comparison with our NotoP gene set, we obtained published microarray (Tamplin et al., 2011; Wymeersch et al., 2019) and RNA-seq datasets (Peck et al., 2017). For the former, we used the provided lists of notochord-specific genes (Tamplin et al.) or genes enriched in E8.5 rostral node and node-streak border relative to remaining E8.5 samples (‘extended spatial analysis’ in Wymeersch et al.). Gene annotations not found in our dataset were converted. For the Tamplin et al. dataset, seven genes (*Defcr-rs2*, *EG639426*, *ENSMUSG00000074335*, *H3073F06*, *LOC672711*, *LOC674134* and *Pkd1l1*), and for the Wymeersch et al. dataset 12 genes (*4930533K18Rik*, *B930045J24Rik*, *Defa1*, *Defcr-rs10*, *Defcr-rs2*, *LOC100040592*, *LOC100044289*, *LOC100046120*, *LOC100048721*, *LOC381284*, *OTTMUSG00000017677* and *scl0003799.1_2*) were not found in our mm10 RefSeq annotation. These genes were removed from the comparative analysis.

The RNA-seq dataset was mapped to chromosomes 1-19, X, Y and M of the mouse mm10 genome using TopHat2 (v2.1.1) and bowtie (v1.2.2) (Kim et al., 2013; Langmead et al., 2009) and the RefSeq annotation in gtf format (UCSC), using the options ‘--no-coverage-search -g1 --library-type fr-firststrand’. FPKM values and differential gene expression were calculated using Cufflinks version 2.2.1 (Trapnell et al., 2013) with the options ‘-u --no-effective-length-correction -b’. We then selected genes with an adjusted *P*-value of <0.01 and a >4-fold increased expression in the notochord of E12.5 embryos compared with nucleus pulposus from postnatal day 0, resulting in 429 differentially expressed genes. The four gene sets were then plotted using nVennR (Perez-Silva et al., 2018).

### qPCR

For the qPCR analysis of different T^mC^/Noto^mT^/Foxa2^EGFP^ populations by FACS, 20,000 cells were sorted for each sample, with the exception of P7 in Dox^+^ d3 (4994 cells), P10 in Dox^+^ d3 (9977 cells) and d3.25 (12,297 cells), and P11 in Dox^+^ d3.5 (15,299 cells). For P11 in Dox^+^ d3 and d3.25, not enough cells were present to be sorted. The RNA was extracted as described for RNA-seq. For cDNA synthesis, 14 μl of each sample was reverse-transcribed using the QuantiTect Reverse Transcription Kit (QIAGEN, 205311) according to the manufacturer's instructions, including the optional increased incubation time of 30 min at 42°C. The resulting cDNA was then diluted with H_2_O, adjusted to the amount of cells that were sorted in each sample. qPCR was performed on a StepOnePlus (Thermo Fisher Scientific) system using the GoTaq qPCR premix (Promega, A6001) according to the manufacturers' recommendations. Each sample and primer combination was loaded in triplicate. Primer sequences are listed in Table S7. Potential residual genomic DNA contamination was first tested using a primer pair detecting genomic DNA in a gene desert region and a melt curve analysis was performed for all reactions to control for potential off-target amplifications. The quantification was performed using StepOne Software v2.3 using the relative quantification (ΔΔCt) method. The −Dox P4 sample at d3 served as the reference sample for all calculations. Expression values were normalized using *Pmm2* was as a reference gene.

### ATAC-seq

ATAC-seq experiments were conducted following an established protocol (Buenrostro et al., 2013). A total of 2000 cells were utilized per ATAC-seq experiment. After FACS sorting, the cells were centrifuged (100 ***g*** for 5 min at 4°C) and the resulting pellets were resuspended in 50 μl of lysis buffer (10 mM Tris pH 7.4, 10 mM NaCl, 3 mM MgCl_2_, 0.1% NP-40) before being subjected to centrifugation at 500 ***g*** at 4°C for 10 min. The supernatant was discarded, and each pellet was then resuspended in the transposition reaction mix (25 μl 2× TD buffer, 2.5 μl Tn5 transposase, 22 μl H_2_O), followed by incubation at 37°C for 30 min. Subsequently, the reaction was halted by adding PB buffer (QIAGEN), and the tagmented DNA was purified using the MinElute kit (QIAGEN). The purified DNA was combined with ATAC index PCR primers and 2× Kapa HiFi Hotstart Readymix and subjected to pre-amplification (98°C for 30 s, followed by eight cycles of 98°C for 10 s, 63°C for 30 s, and 72°C for 1 min) in a 50 μl reaction volume. To determine the optimal number of additional cycles and prevent overamplification, 5 μl of the pre-amplification mix was combined with primers, 1× Evagreen Sybr green (Jena Biosciences), and 2× Kapa HiFi Hotstart Readymix in a 15 μl total volume and subjected to 30 cycles on a StepOne Plus instrument. The remaining pre-amplified samples (45 μl) were subjected to an additional seven (NotoP sample) or six (MP sample) cycles, resulting in a total of 15 (NotoP sample) or 14 (MP sample) cycles. The libraries were purified using MinElute columns (QIAGEN), and the concentration was determined using the DNA HS Qubit assay (Life Technologies). Approximately 4 ng of each library was subjected to DNA HS Bioanalyzer chip (Agilent) to assess library size and calculate molarities. The samples were pooled and subjected to paired-end sequencing on an Illumina NovaSeq 6000 platform with 2×100 bp read length.

### ChIP-seq

For histone ChIP-seq on sorted NotoP^diff^ and MP^diff^ cells, the iDeal ChIP-Seq kit (Diagenode) was used according to the manufacturer's instructions. Approximately 200,000 cells were used per ChIP. ChIP on bulk d4 *in vitro*-differentiated notochord-like cells for the identification of T- and Foxa2-binding sites was performed as described previously (Koch et al., 2011). Antibodies are listed in Table S8. ChIP-Seq sequencing libraries were generated using the TrueSeq ChIP-Seq kit (Ilumina) following the manufacturer's instructions with minor modifications. After adapter ligation, 0.95× of AMPure XP beads (Beckman Coulter, A63880) were used for a single purification and the DNA was eluted using 15 µl of resuspension buffer (RSB, Illumina). After the addition of 1 µl primer mix (25 mM each; Primer 1: 5′-AATGATACGGCGACCACCGA*G-3′; Primer 2: 5′-CAAGCAGAAGACGGCATACGA*G-3′) and 15 µl 2× Kapa HiFi HotStart Ready Mix (Kapa Biosystems), amplification was performed for 45 s at 98°C; five cycles of 15 s at 98°C, 30 s at 63°C and 30 s at 72°C; and a final 1 min incubation at 72°C. The PCR products were purified using 0.95× of beads and eluted using 21 µl of RSB. Libraries were directly amplified for an additional 13 cycles and purified using AMPure XP beads. The libraries were quantified using the Qubit DNA HS assay and the library size was validated using DNA HS bioanalyzer chips (Agilent, 5067-4626). The samples were pooled and subjected to paired-end sequencing on an Illumina NextSeq 500 platform with 2×75 bp read length.

Reads were mapped to chromosomes 1-19, X, Y and M of the mouse mm10 genome using bowtie version 1.3.1 (Langmead et al., 2009), providing the options ‘ -y -m 1 -S -I 100 -X 500’. The mapping information of the paired-end reads was used to elongate each fragment to its original size using a custom perl script, with the result stored as a BED file. Reads were then sorted and deduplicated such that only one fragment with the same starting and end position was retained. For visualization, wiggle files were generated with BEDTools version 2.23.0 (Quinlan and Hall, 2010), converted to bigwig format and analyzed in the Integrated Genome Browser (Freese et al., 2016).

Peak detection was performed using MACS version 3.0.0b1 (https://github.com/macs3-project/MACS) using the elongated and deduplicated bed files as inputs and setting a q-value cutoff of 0.1.

### ATAC-seq data processing

For ATAC-seq mapping, adapters were detected and removed using fastq-mcf of ea-utils (https://github.com/ExpressionAnalysis/ea-utils, version 1.04.738 was used) and mapped to chromosomes 1-19, X, Y and M of the mouse mm10 using bowtie (Langmead, et al., 2009) version 1.3.1), with the options ‘-y -m 1 -S -X 2000 --allow-contain’. Mapped paired-end reads were converted to a bed file by generating the original fragment using a custom perl script and duplicates were removed such that only one fragment with the same starting and end position was retained. Owing to the repetitive nature of the Y chromosome and non-informative mitochondrial genome, reads mapped to either of them were removed and .wig files were generated using BEDTools and converted to bigwig format.

### Differential ATAC-seq analysis

In order to identify regions with differential accessibilities between the NotoP and MP samples, we employed diffReps version 1.55.4 (Shen et al., 2013), using the elongated and deduplicated ATAC-NotoP and ATAC-MP samples as treatment and control, respectively. We used the default parameters, with the exception of performing the statistical analysis using a G-test (‘--meth gt’) and disabling the DNA fragment shifting (‘--frag 0’) owing to the use of already elongated reads. This resulted in 16,680 regions that displayed either a significantly higher (more open in NotoPs) or lower (more open in MPs) accessibility. We then removed all those regions overlapping known promotors (mm10 UCSC refseq genes), defined as ±5 kb from known transcription start sites, resulting in 13,890 differential regions.

### Transcription factor peaks overlaps

In order to avoid inclusion of transcription factor peaks that fall within regions displaying mapping artifacts in ATAC-seq data, we removed all peaks falling within those 35 previously identified regions (Koch et al., 2017; Buenrostro et al., 2015). We then overlapped the peak regions of the T-NMPdiff, T-NotoPdiff and Foxa2-NotoPdiff datasets to obtain all seven possible combinatorial binding profiles. We then used the single bp maxima of each peak and intersected those with ATAC regions displaying a significant increase in accessibility in NotoP cells. Finally, each peak was assigned potential target genes depending on the binding location. Intergenic peaks (at least 5 kb away from any gene annotation) were assigned to both the closest up- and downstream gene, whereas genic peaks (those located between −5 kb of the promoter and +5 kb after the gene end) were assigned to that gene (or multiple in case of overlapping genes) as well as the closest up- and downstream gene.

### Heatmaps and average profiles

Heatmaps and average profiles were generated using SeqMINER (Ye et al., 2011). For plotting the ATAC-seq NotoP versus MP comparison, the reference bed files were generated by using the center of differential ATAC regions and sorting them by the generated diffReps *P*-values. For plotting the T, Foxa2, H3K27ac and ATAC profiles, we used the peak maxima of T-NMP^diff^, T-NotoP^diff^ or Foxa2-NotoP^diff^ (in that respective order if present) and sorted the peaks first by the seven different combinations and then randomized the peaks within each category. The corresponding elongated and deduplicated bed files were used as inputs and hence set the extension size to 0.

### UpSet plot

The UpSet plot was generated using UpSetR (Conway et al., 2017) to visualize associations between peak categories and associated genes.

### Note added in proof

While this work was prepared for publication, complementary work dissecting the regulatory landscape of the *T* locus with respect to notochord control elements was reported on the bioRxiv preprint website (Kemmler et al., 2023 preprint). In this work, the notochord enhancers *TNE*, *TNE2* and *TE7* were termed *T3*, *C* and *I*, respectively. The genomic fragments defining these enhancers are different from ours, but with considerable overlaps.

## Supplementary Material

10.1242/develop.202111_sup1 Supplementary information

Table S1. Relative Expression values (FPKM) for RNA-seq samples.

Table S2. Intersections of notochord gene sets shown in Figure S2C.

Table S3. List of the 3728 candidate enhancers identified by differential ATAC-seqand TF binding analysis.

Table S4. List of the 319 notochord genes with and number of associated enhancercategories.
