# Supplementary material for: Genome-wide identification of notochord enhancers comprising the regulatory landscape of the brachyury locus in mouse
Source: Development. 2023 Nov 9;150(22):dev202111. doi: 10.1242/dev.202111 (PMC10651091; doi:10.1242/dev.202111)
Supplement: Supplementary information [file develop-150-202111-s1.pdf]

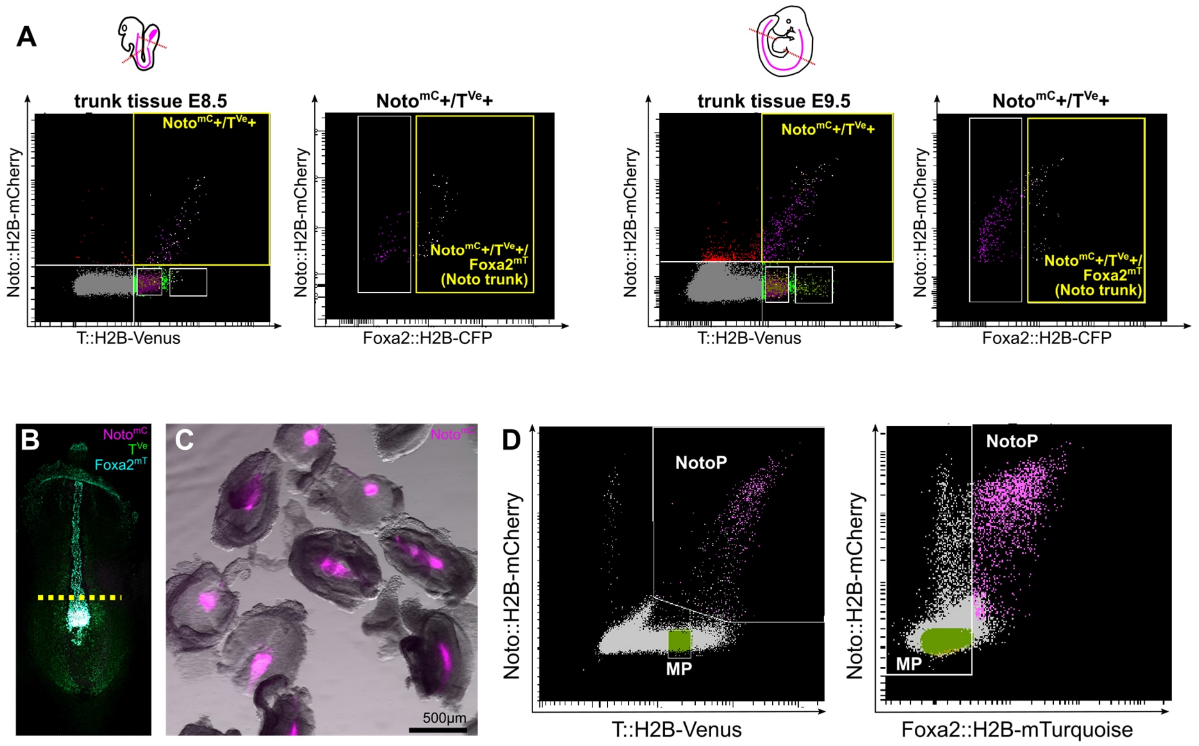


**Fig. S1. Generation of ATAC-seq data from subpopulations from E8.5 caudal ends.(A)** FACS profile with subpopulations based on Noto$^{mC}$/T$^{Ve}$/Foxa2$^{mT}$ reporter expression used for sorting of subpopulations for transcriptome analysis of trunk notochord (Noto trunk). **(B-D)** Workflow for sample preparation and FACS for ATAC-seq. **(B)** Maximum intensity projection of a E8.5 embryo generated from the Noto$^{mC}$/T$^{Ve}$/ Foxa2$^{mT}$ mESC line. Caudal ends were dissected from E8.5 embryos at the somite border indicated by the yellow dotted line. **(C)** Isolated caudal ends used for the experiment. **(D)** FACS profile with subpopulations based on Noto$^{mC}$/T$^{Ve}$/Foxa2$^{mT}$ reporter expression.

Development • Supplementary information

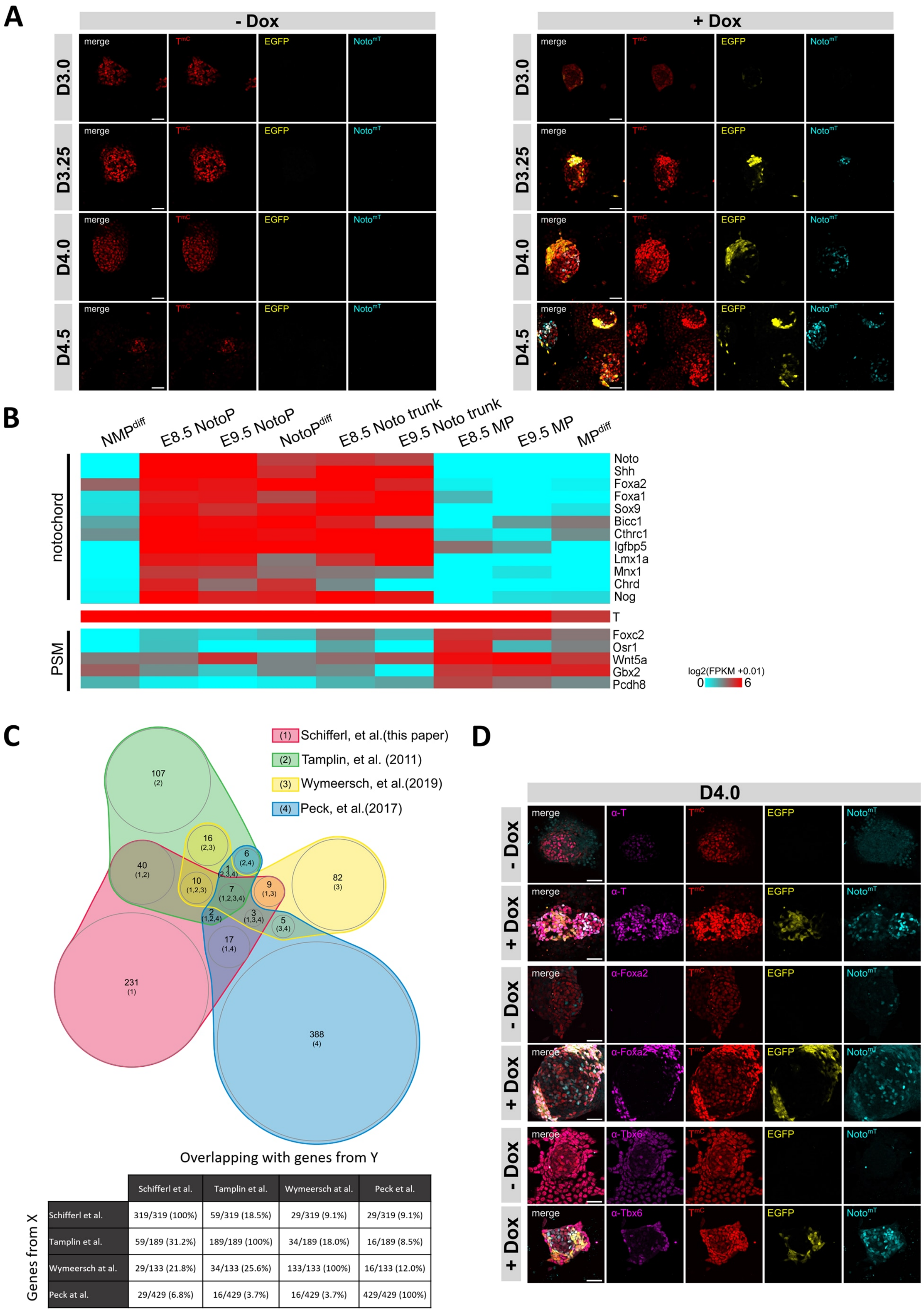


| | Schifferl et al. | Tamplin et al. | Wymeersch at al. | Peck et al. |
|---|---|---|---|---|
| Schifferl et al. | 319/319 (100%) | 59/319 (18.5%) | 29/319 (9.1%) | 29/319 (9.1%) |
| Tamplin et al. | 59/189 (31.2%) | 189/189 (100%) | 34/189 (18.0%) | 16/189 (8.5%) |
| Wymeersch at al. | 29/133 (21.8%) | 34/133 (25.6%) | 133/133 (100%) | 16/133 (12.0%) |
| Peck at al. | 29/429 (6.8%) | 16/429 (3.7%) | 16/429 (3.7%) | 429/429 (100%) |

**Fig. S2. Validation of *in vitro* derived NotoPs.**

Development • Supplementary information

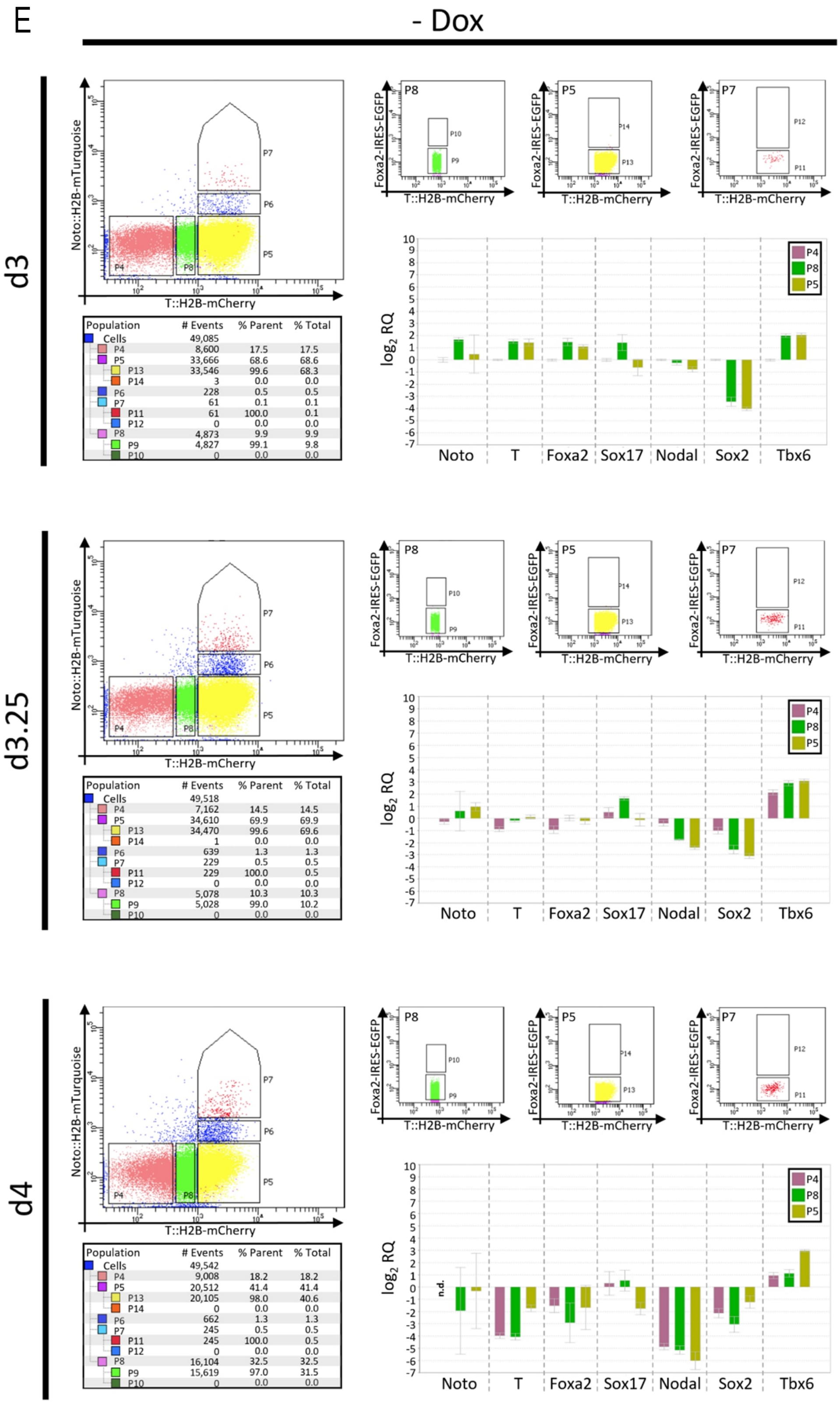


**Fig. S2. Validation of *in vitro* derived NotoPs.**

Development • Supplementary information

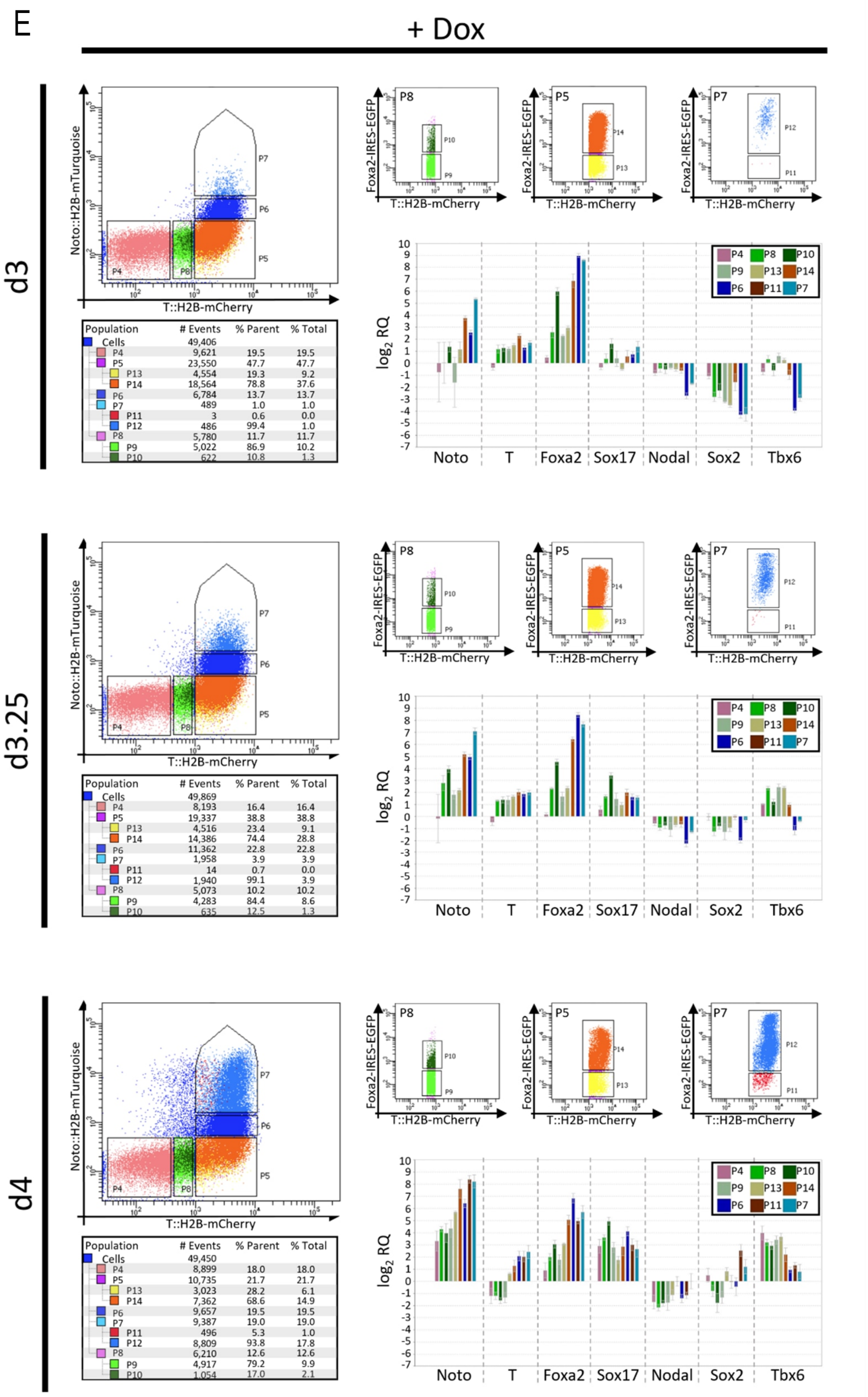


**Fig. S2. Validation of *in vitro* derived NotoPs.**

Development • Supplementary information

**Fig. S2. Validation of *in vitro* derived NotoPs.**

**(A**) Images of differentiated colonies acquired by confocal microscopy without and with doxycycline (Dox) induced *Foxa2* expression. Scale bar = 50 µm. **(B)** Log transformed FPKM values of selected marker genes across analyzed samples. **(C)** Top: Venn diagram showing the intersections between the NotoP gene set determined in this study and gene sets from previously published studies. Tamplin et al (2011): 189 genes enriched in E8.5 Noto-GFP+ vs. Noto-GFP- cells (microarray). Wymeersch et al (2019): 133 genes enriched in E8.5 node streak border and rostral node vs. remaining samples (microarray). Peck et al (2017): 429 genes enriched in E12.5 Shh-Cre+ notochord vs. P0 Shh-Cre+ nucleus pulposus (RNA-seq). Bottom: Table showing the pairwise overlap of datasets. The genes in the individual groups are listed in Supplementary Table S2. **(D)** Images of differentiated colonies with immunofluorescence for T, Foxa2 or Tbx6 acquired by confocal microscopy. Scale bar = 50 µm. **(E)** Time course FACS profiles and qPCR for marker genes on cDNA isolated from sorted subpopulations during differentiation with and without Dox-induction. $NMP^{diff}$, $MP^{diff}$ or $NotoP^{diff}$ cells used for ChIP-seq/RNA-seq experiments are derived from cell populations P5 d3.0 -Dox, P5 d4.0 -Dox, or P7 d4.0 +Dox, respectively. The $NotoP^{diff}$ cell number of P7 (+Dox) is increasing from d3 to d4 and characterized by increasing Noto expression as well as high T and Foxa2 expression. The $NMP^{diff}$ cell population P5 (d3, -Dox) is decreasing in number and differentiating to $MP^{diff}$ (P5, -Dox) on d4, characterized by increasing *Tbx6* and parallel decreasing *T* expression. *Nodal* expression decreases in all cell populations, however reaching initial levels again in P7 at d4. *Sox17* expression is highest in P10 (d4, +Dox), which might represent a small subpopulation of endodermal cells (2.1% of total). All qPCR data were calculated relative to the expression level of the respective gene in $T^{Ve}$ negative cells (d3, -Dox). Error bars indicate the 95% confidence interval of 2-3 technical replicates.

Development • Supplementary information

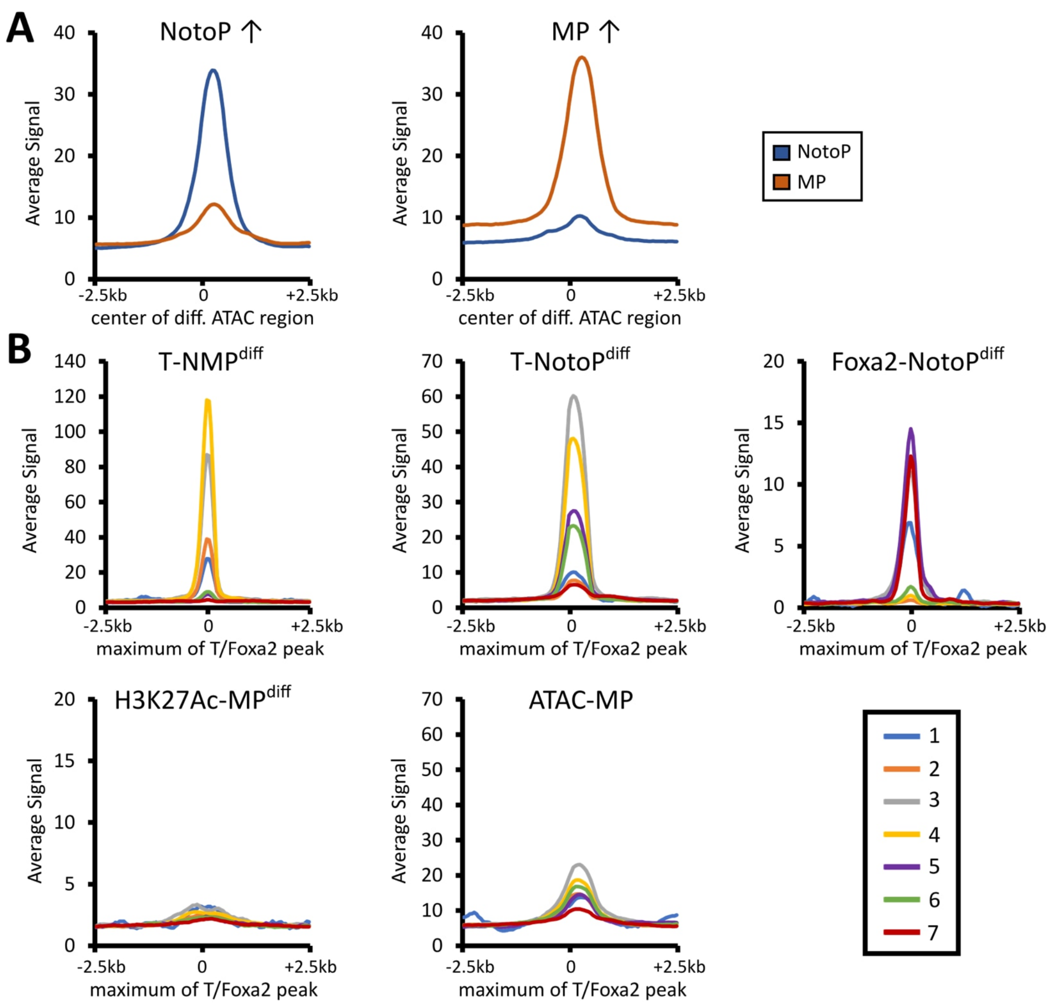


**Fig. S3. Average Profiles genome-wide ATAC- and ChIP-seq datasets.**

(A) ATAC-seq signal in NotoP (blue) and MP (orange) cells around differential ATAC regions displaying higher accessibility in NotoP (left) or MP (right) cells. (B) Remaining average profiles of ATAC- and ChIP-seq datasets for the different enhancer categories.

Development • Supplementary information

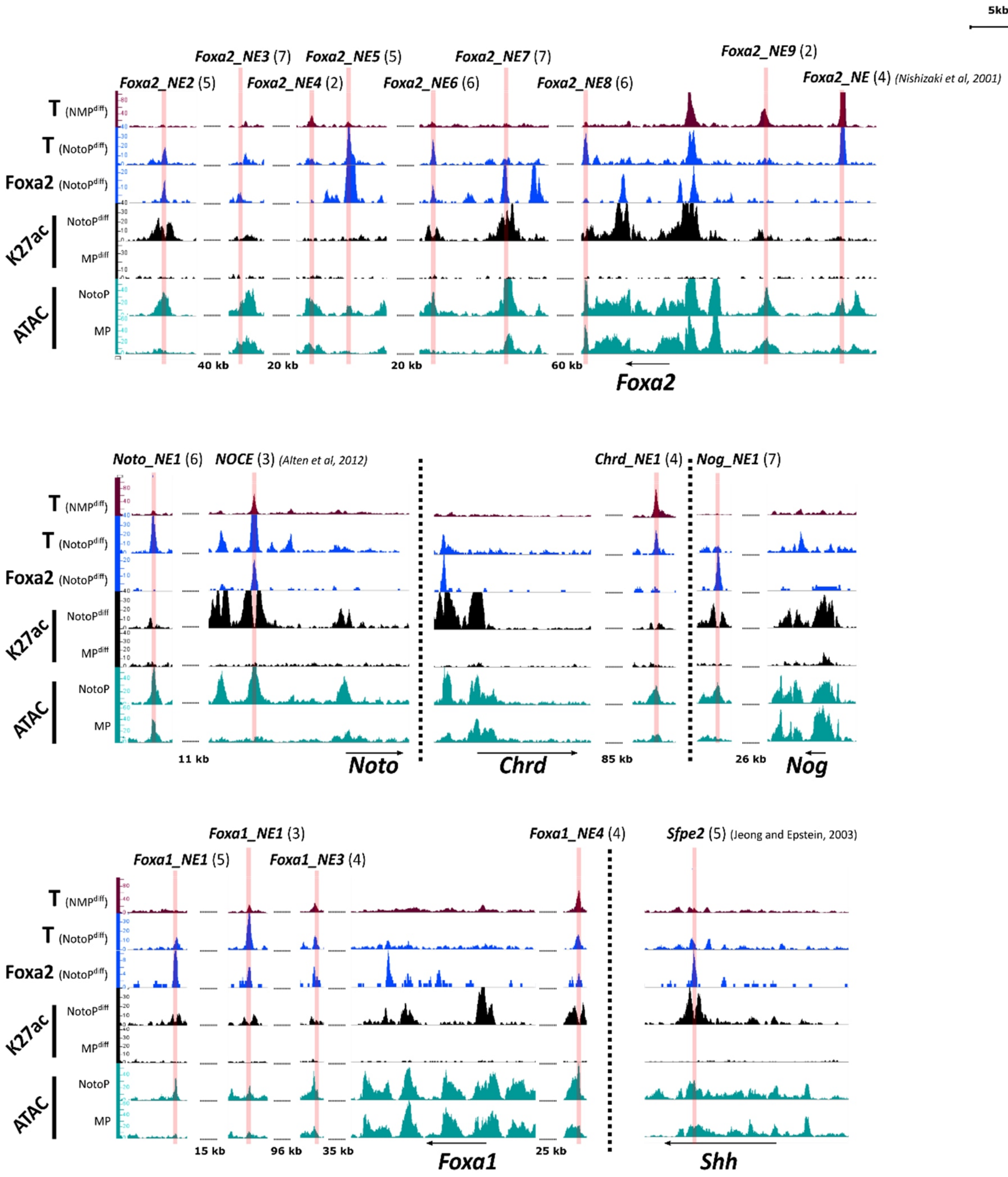


**Fig. S4. Genome browser screenshots of identified enhancers at important notochord genes.**

Genome browser snapshots showing loci of notochord genes with tracks for TF ChIP-seq, H3K27ac ChIP-seq and ATAC-Seq. Track maxima are normalized to the number of mapped reads for each antibody and ATAC experiment. Elements identified in this study are highlighted in red. The number in brackets indicates the enhancer category as shown in Fig.2B.

Development • Supplementary information

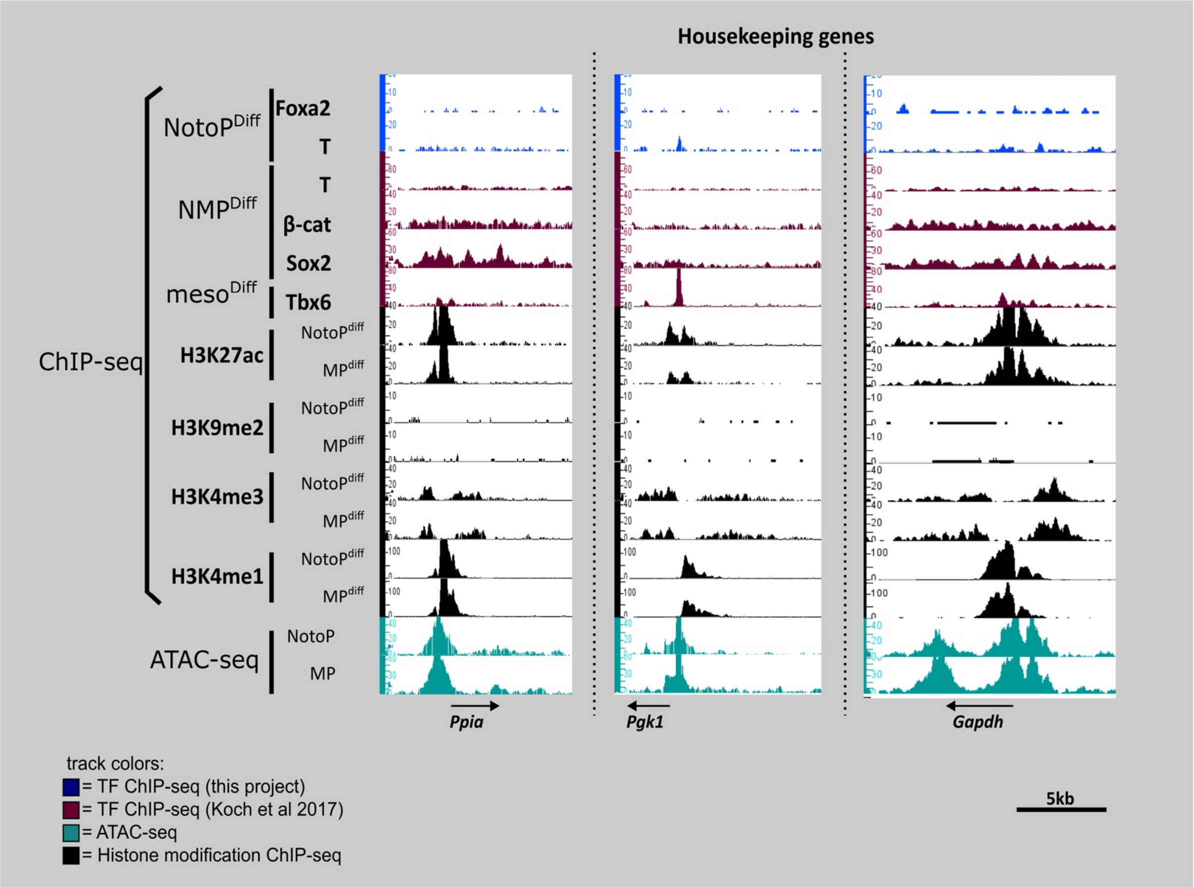


**Fig. S5. Genome browser screenshots of exemplary housekeeping loci.**

Genome browser snapshots showing loci of housekeeping genes with tracks for TF ChIP-seq, Histone 3 ChIP-seq and ATAC-seq. Track maxima are normalized to the number of mapped reads for each antibody and ATAC experiment. The same normalization was used in Figures 3,4, S4 and S5.

Development • Supplementary information

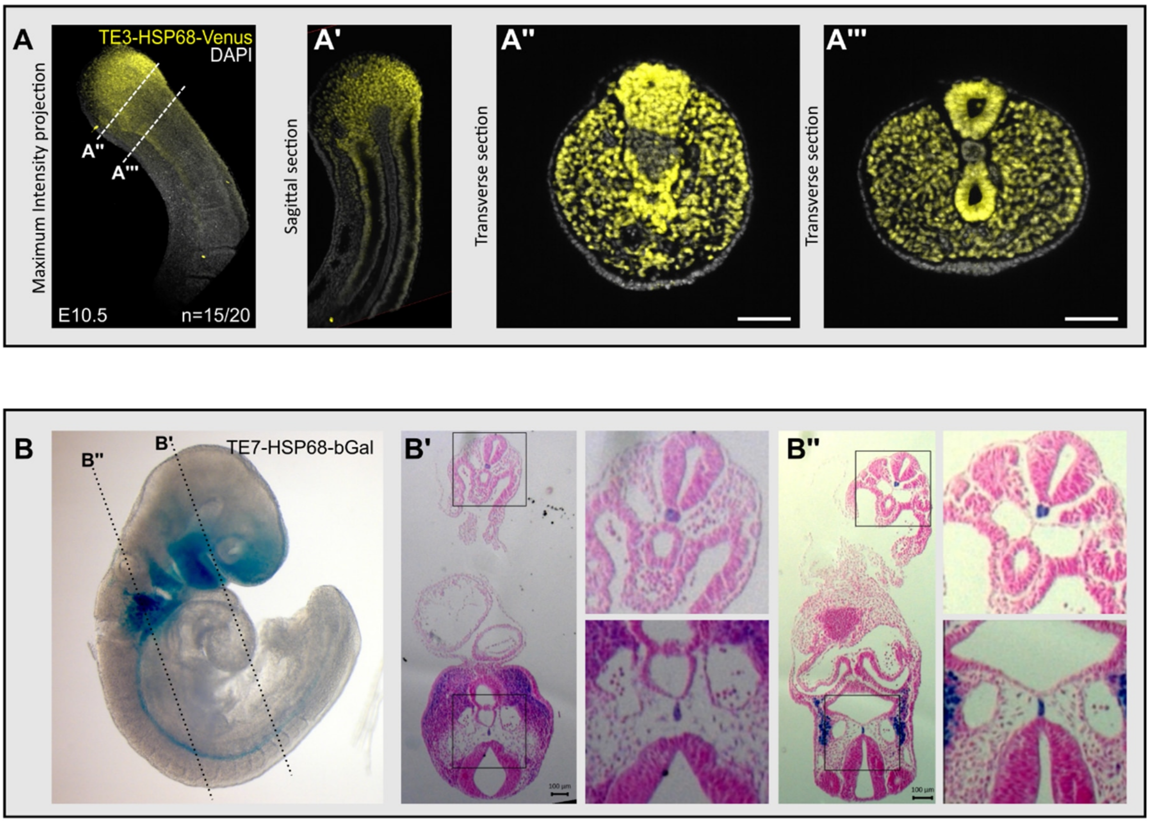


**Fig. S6. Activity assays of enhancer elements in the murine *T* locus.**

**(A)** Light sheet micrograph of *TE3*-driven Venus reporter expression in the tail bud of a E10.5 embryo. Nuclei stained with DAPI (grey). Left: maximum intensity projection. **(A')** Sagittal optical midline section showing activity in paraxial mesoderm, posterior neural tube and hindgut pocket. **(A''-A''')** Transverse optical sections acquired by light sheet microscopy at the axial levels indicated in (A). Scale bar = 100µm. **(B)** *TE7*-driven galactosidase activity visualized in a E9.25 embryo. Dotted lines indicate the axial position of transverse histological sections with eosin staining shown in **(B'-B'').**

Development • Supplementary information

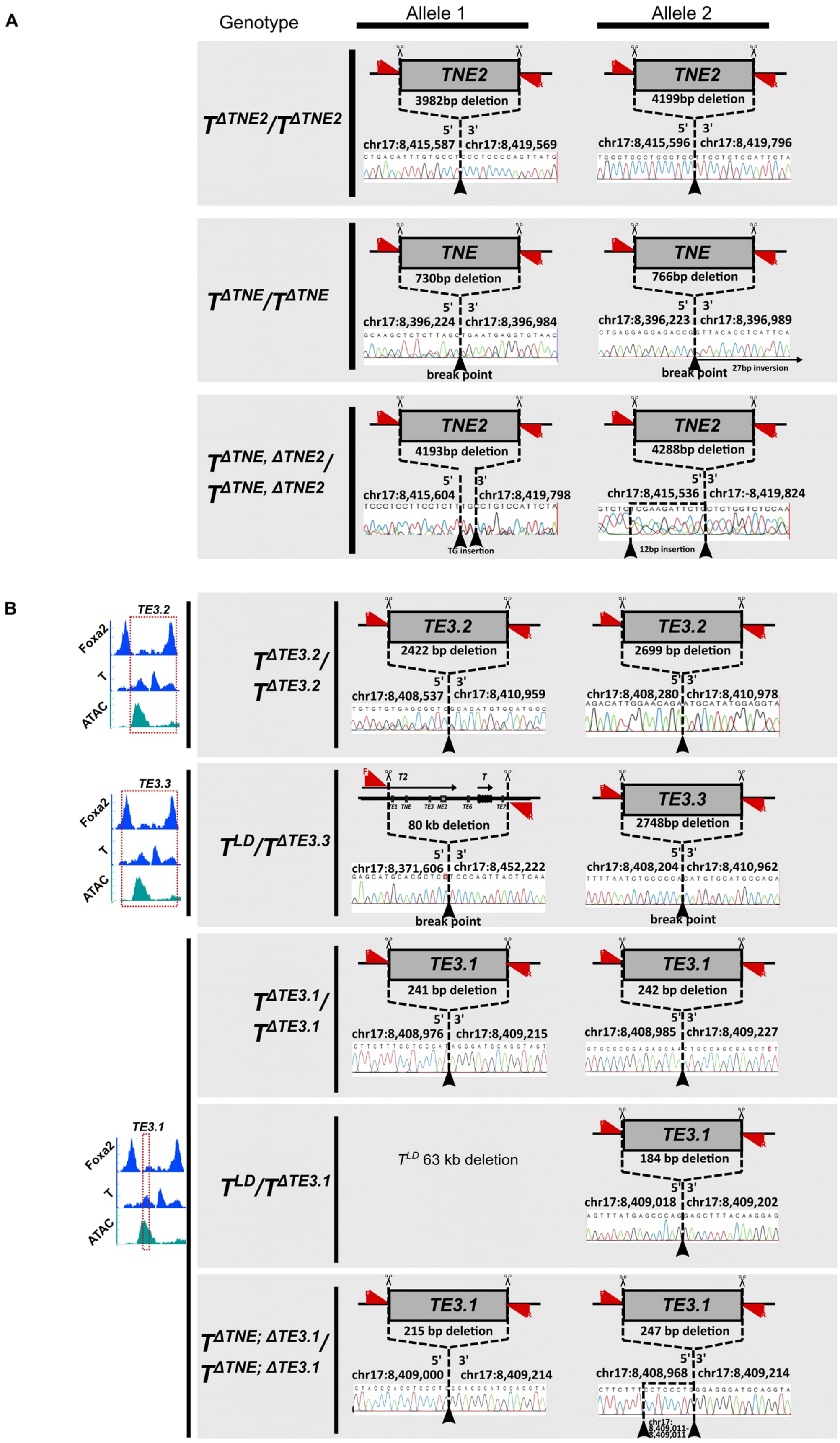


**Fig. S7. Genotyping of Deletions**

Development • Supplementary information

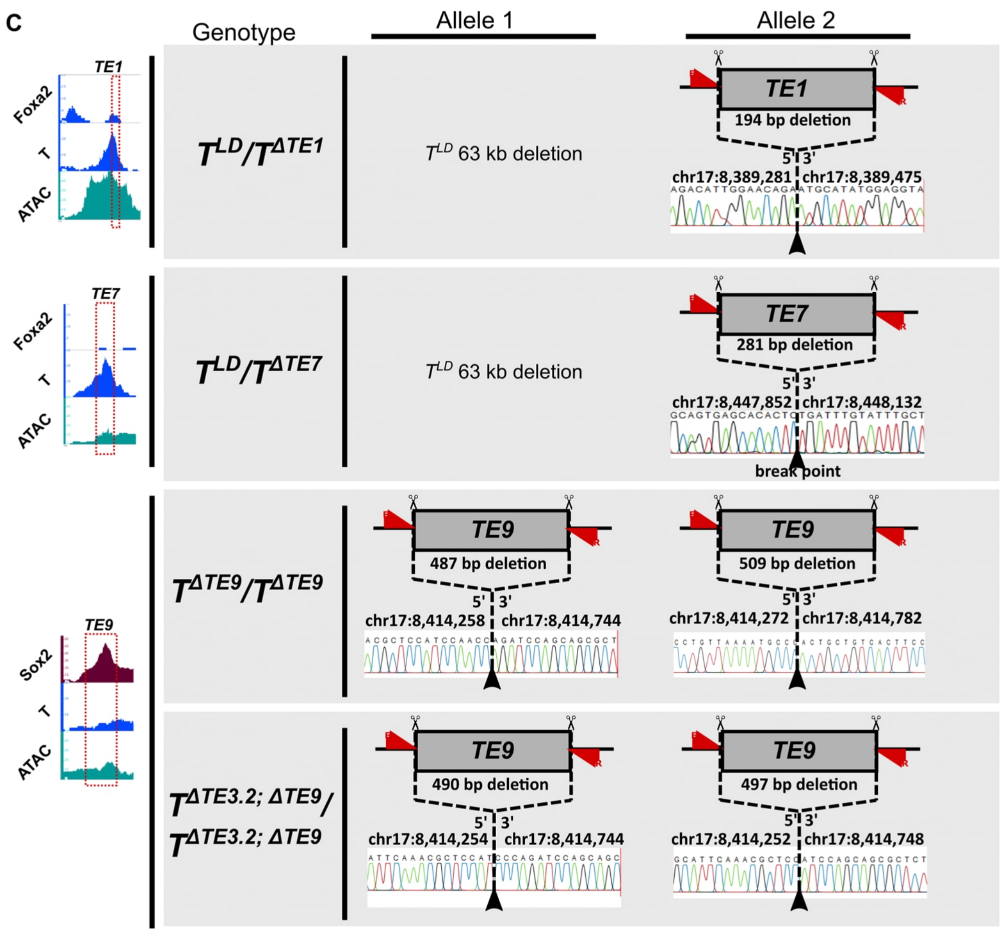


**Fig. S7. Genotyping of Deletions**

Red arrows indicate the position of the forward (F) and reverse (R) primers used for genotyping by PCR, not to scale. Scissors icons indicate the approximate positions of gRNA target sites. The dotted lines converging at the break points mark the CRISPR/Cas9 mediated deletions. The coordinates (mm10) of the bordering 5' and 3' edges of the respective deletions are specified next to the break point(s). Sanger sequencing tracks of enhancer mutant PCR fragment or subcloned PCR fragments in case of double deletions show the region flanking the deletions. For each genotype at least two clones were generated and checked for phenotypic identity. **(A)** TNE and TNE2 mutants shown in Main Figure 4. **(B)** TE3 mutants. **(C)** TE1, TE7 and TE9 mutants. The $T^{LD}$ mutant was reported previously (Schifferl et al, 2021).

Development • Supplementary information

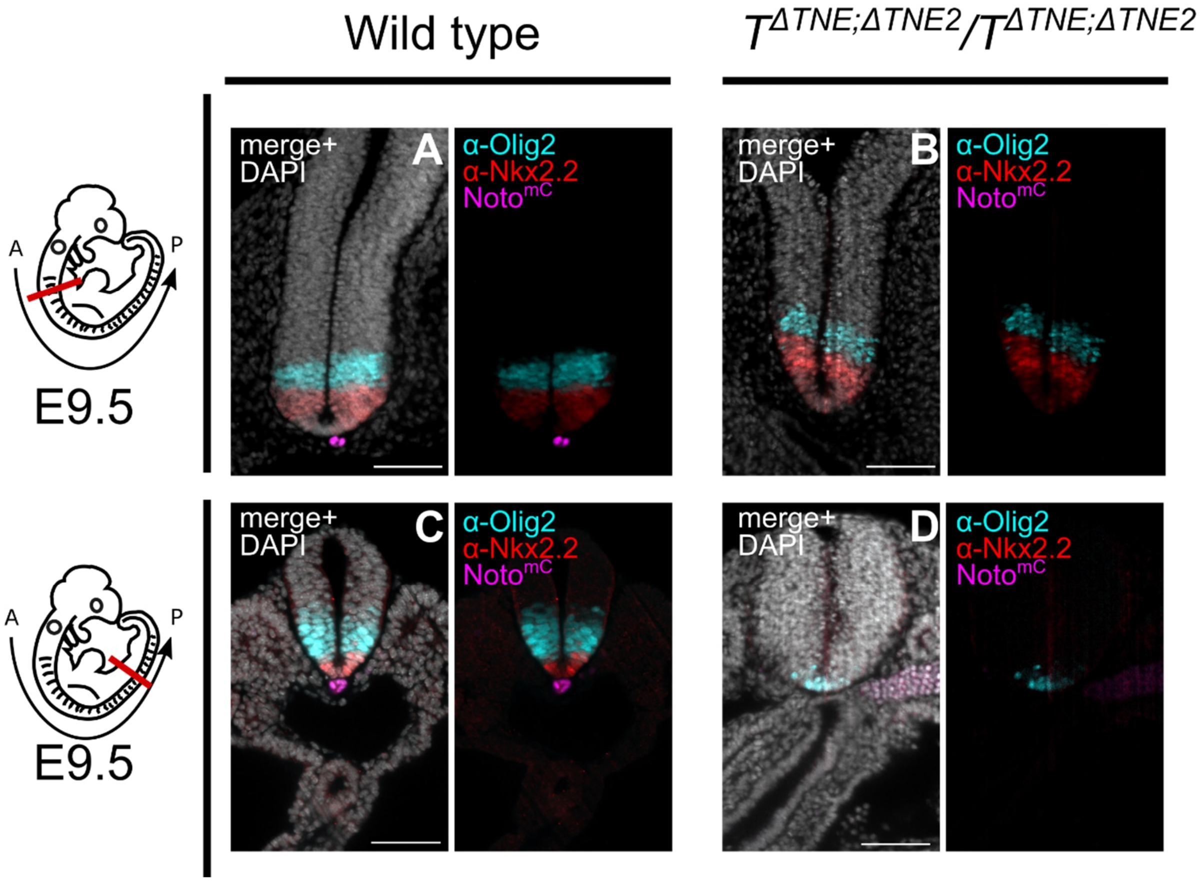


**Fig. S8. Loss of trunk notochord in *TNE; TNE2* double enhancer mutants leads to neural tube patterning defects.**

Light sheet micrographs of wild type and mutant embryos with immunofluorescence for Olig2 (blue) and Nkx2.2 (red). Transversal optical sections at different axial positions at E9.75 as indicated in the schematic on the left. **(A-B)**: Cervical. **(C-D)**: Lumbar. Scale bar = 100 µm.

Development • Supplementary information

**Table S1. Relative Expression values (FPKM) for RNA-seq samples.**

Available for download at
https://journals.biologists.com/dev/article-lookup/doi/10.1242/dev.202111#supplementary-data

**Table S2. Intersections of notochord gene sets shown in Figure S2C.**

Available for download at
https://journals.biologists.com/dev/article-lookup/doi/10.1242/dev.202111#supplementary-data

**Table S3. List of the 3728 candidate enhancers identified by differential ATAC-seq and TF binding analysis.**

Available for download at
https://journals.biologists.com/dev/article-lookup/doi/10.1242/dev.202111#supplementary-data

**Table S4. List of the 319 notochord genes with and number of associated enhancer categories.**

Available for download at
https://journals.biologists.com/dev/article-lookup/doi/10.1242/dev.202111#supplementary-data

## Table S5. Cell lines used in this work

| modification | parental clone | construct | transfection method | selection | purpose | published |
|---|---|---|---|---|---|---|
| $Noto^{mC}$ | F1G4 (George et al., 2007 | Noto::H2B-mCherry-pA-FRT-PGK-hygro-pA-FRT BAC | electroporation | hygromycin (150µg/ml) | wild type reference for mutant analyis | Schifferl et al 2021 |
| $Noto^{mC}/T^{Ve}/Foxa2^{mT}$ | $Noto^{mC}$ | T::H2B-Venus-pA-FRT-PGK-neo-pA-FRT BAC Foxa2::H2B-mTurquoise-pA-FRT-PGK-puro-pA-FRT BAC | electroporation | G418 (250µg/ml, puromycin (1µg/ml) | Derivation of subpopulations for RNA-seq | this study |
| $T^{\Delta TNE2}/T^{\Delta TNE2}$ | $Noto^{mC}$ | px459-NE2-g1 (transient) px459-NE2-g2 (transient) | lipofectamine | puromycin (d1-2 2µg/ml; d3 1µg/ml) | generation of enhancer mutant alleles | this study |
| $T^{\Delta TNE}/\ T^{\Delta TNE}$ | $Noto^{mC}$ | px459-TNE-g1 (transient) px459-TNE-g2 (transient) | lipofectamine | puromycin (d1-2 2µg/ml; d3 1µg/ml) | generation of enhancer mutant alleles | Schifferl et al 2021 |
| $T^{\Delta TNE;\ \Delta TNE2}/\ T^{\Delta TNE;\ \Delta TNE2}$ | $T^{\Delta TNE}/\ T^{\Delta TNE}$ | px459-NE2-g1 (transient) px459-NE2-g2 (transient) | lipofectamine | puromycin (d1-2 2µg/ml; d3 1µg/ml) | generation of enhancer mutant alleles | this study |
| $R26^{RMC}$ (Vidigal et al 2010) | F1G4 (George et al., 2007 | loxP-PGK-Hygro-pA-loxP-neo-pA | electroporation | hygromycin (150µg/ml) | parental | Vidigal et al 2010 |
| *TRE::Foxa2* | $R26^{RMC}$ | pDonor-loxP-TRE-Foxa2-IRES-EGFP-pA-PGK-loxP PGK-Cre | lipofectamine | G418 (350µg/ml) | Foxa2 OE line | this study |
| $TRE::Foxa2/T^{mC\text{-}2A\text{-}rtTA}$ | *TRE::Foxa2* | T::H2B-mCherry-T2A-irTA-pA-FRT-PGK-puro-pA-FRT BAC | electroporation | puromycin (1µg/ml) | driver for inducible Foxa2 OE | this study |
| $TRE::Foxa2/T^{mC\text{-}2A\text{-}rtTA}/Noto^{mT}$ | $TRE::Foxa2/\ T^{mC\text{-}2A\text{-}rtTA}$ | Noto::H2B-mTurquoise-pA-FRT-PGK-Bla-pA-FRT BAC | electroporation | blasticidin (5µg/ml) | purification of NotoPs | this study |
| *TNE2-HSP68-Venus* | $R26^{RMC}$ | pDonor-loxP-NE2-HSP68-Venus-pA-PGK-loxP; PGK-Cre (transient) | lipofectamine | G418 (350µg/ml) | enhancer activity assay | this study |
| *TE3-HSP68-Venus* | $R26^{RMC}$ | pDonor-loxP-TE3-HSP68-Venus-pA-PGK-loxP; PGK-Cre (transient) | lipofectamine | G418 (350µg/ml) | enhancer activity assay | this study |
| *TE7-HSP68-bGal* | $R26^{RMC}$ | pDonor-loxP-TE7-HSP68-bGal-pA-PGK-loxP; PGK-Cre (transient) | lipofectamine | G418 (350µg/ml) | enhancer activity assay | this study |

Development • Supplementary information

**Table S6. Mutant phenotypes of enhancer knockout lines shown in Fig. S7-8**

| genotype | deletion (mm10) | size | E9.5 notochord phenotype | E11-12.5 tail outgrowth | embryos analyzed |
|---|---|---|---|---|---|
| $T^{\Delta TE3.2}/T^{\Delta TE3.2}$ | chr17:8,408,537-8,410,959 | 2422 | normal | yes | 16 (E11.5) |
| | chr17:8,408,280-8,410,978 | 2699 | | | |
| $T^{LD}/T^{\Delta TE3.3}$ | chr17:8,371,606-8,452,222 | 80617 | like parental clone ($T^{LD}$) | yes | 10 (E11.5) |
| | chr17:8,408,204-8,410,962 | 2748 | | | |
| $T^{\Delta TE3.1}/T^{\Delta TE3.1}$ | chr17:8,408,976-8,409,216 | 241 | normal | yes | 8 (E10.5)<br>4 (E12.5)<br>4 (E12.5) |
| | chr17:8,408,985-8,409,227 | 242 | | | |
| $T^{LD}/T^{\Delta TE3.1}$ | chr17:8,389,017-8,451,946 | 63kb | like parental clone ($T^{LD}$) | yes | 10 (E9.5)<br>4 (E11.5)<br>5 (E9.5)<br>4 (E12.5) |
| | chr17:8,409,018-8,409,204 | 184 | | | |
| $T^{\Delta TNE;\ \Delta TE3.1}/T^{\Delta TNE;\ \Delta TE3.1}$ | chr17:8,409,000-8,409,214 | 215 | like parental clone ($T^{LD}$) | yes | 11 (E9.5)<br>12 (E11.5) |
| | chr17:8,408,968-8,409,214 | 247 | | | |
| $T^{LD}/T^{\Delta TE1}$ | chr17:8,389,017-8,451,946 | 63kb | like parental clone ($T^{LD}$) | yes | 7 (E9.5)<br>3 (E10.5) |
| | chr17:8,389,281-8,389,475 | 194 | | | |
| $T^{LD}/T^{\Delta TE7}$ | chr17:8,389,017-8,451,946 | 63kb | like parental clone ($T^{LD}$) | yes | 11 (E8.5)<br>10 (E9.5)<br>5 (E10.5)<br>1 (E12.5) |
| | chr17:8,389,017-8,451,946 | 281 | | | |
| $T^{\Delta TE9}/T^{\Delta TE9}$ | chr17:8,414,258-8,414,744 | 487 | normal | yes | 25 (E11.5) |
| | chr17:8,414,273-8,414,782 | 509 | | | |
| $T^{\Delta TE3;\Delta TE9}/T^{\Delta TE3;\Delta TE9}$ | chr17:8,414,254-8,414,744 | 490 | normal | yes | 9 (E9.5) |
| | chr17:8,414,252-8,414,749 | 497 | | | |

Development • Supplementary information

# Table S7. List of oligonucleotides used for cloning and PCR

| Name | 5'-3' Sequence | Purpose |
|---|---|---|
| TNE2_fw | GTGGCCTGTCAGCTTTGTC | $T^{\Delta TNE2}$ CRISPR genotyping |
| TNE2_R | CATGTGTGTATCTGTGCG | $T^{\Delta TNE2}$ CRISPR genotyping |
| TNE2_g1_top | CACCGCCATCTCCCATTTCCGGAAG | $T^{\Delta TNE2}$ CRISPR genotyping |
| TNE2_g1_bot | AAACCTTCCGGAAATGGGAGATGGC | $T^{\Delta TNE2}$ CRISPR genotyping |
| TNE2_g2_top | caccgTCTAGAATGGACAGGAACGC | $T^{\Delta TNE2}$ CRISPR genotyping |
| TNE2_g2_bot | aaacGCGTTCCTGTCCATTCTAGAc | $T^{\Delta TNE2}$ CRISPR genotyping |
| TNE2_fw_BamHI | TTTTTGGATCCGTGGCCTGTCAGCTT TGTC | Enhancer reporter cloning |
| TE3_fw_BamHI | TTTTTGGATCCCGCGTCCTCAGCCTT TAC | Enhancer reporter cloning |
| TE3_rev_BamHI | TTTTTCCTAGGGGTTATCAGCCCTCC TCCTG | Enhancer reporter cloning |
| TE7_BamHI_fw_new | TTTTTGGATCCCACTCTGTCATGTGG CCC | Enhancer reporter cloning |
| TE7_BamHI_rv_new | TTTTTCCTAGGCAAATCACCGTGCAA GCC | Enhancer reporter cloning |
| T_5_homology_H2B_F | TGTTGGGTAGGGAGTCAAGACTCCT GGAAGGTGGAGAGGGTGGCGGGAG GATGCCAGAGCCAGCGAAGTC | BAC reporter recombineering |
| T_3_homolgy_FRT_R | TCCACTCGGTACTGCAGGCTCTTCC CTGCGCTCTCTGTGCCCGGCGAGCT ATTATGTACCTGACTGATGAAGTTCC | BAC reporter recombineering |
| T_geno_F | GAAGGTGGCTGTTGGGTAGG | BAC reporter genotyping |
| T_geno_R | TCGCAGTTCGCGTTCGGTGG | BAC reporter genotyping |
| H2B-Venus_R | GGAATAGCTCTCCTTGCGGC | BAC reporter genotyping |
| Noto-5_homology_H2B_F | CTCCCATTGAGCTCCTTGCACAGCCT GGGAGGTCCCCTCAGGGTCGCGCA ATGCCAGAACCAGCCAAATC | BAC reporter genotyping |
| Noto_3_homolgy_FRT_R | GGGCGCAGGCTCCCGGGCTGGACC TGAGTGCCTGAGGGAGCAGGGCTG GATTCCCAGTCACGACGTTGTA | BAC reporter recombineering |
| Noto_geno_F | GGCCTCAATCAGCGATGATTAAG | BAC reporter genotyping |
| Noto_geno_R | CTGGACCTGAGTGCCTGAG | BAC reporter genotyping |
| Pmm2_F | CCCCTTTCTGAAGCACTCTG | qPCR |
| Pmm2_R | TAAGGCGTCATTTCCCAAAG | qPCR |
| Noto_F | ATGTCACTCACCACCAGCAG | qPCR |
| Noto_R | CAGCTGCTGCAAGTTAAACG | qPCR |
| T_F | AACTGGTCTAGCCTCGGAGT | qPCR |
| T_R | CTCACAGACCAGAGACTGGG | qPCR |
| Foxa2_F | CGAGCACCATTACGCCTTCAAC | qPCR |
| Foxa2_R | AGTGCATGACCTGTTCGTAGGC | qPCR |
| Sox17_F | AGCCATTTCCTCCGTGGTGT | qPCR |
| Sox17_R | AACACTGCTTCTGGCCCTCAG | qPCR |
| Nodal_F | GGTGGACTTCAACCTGATTGGC | qPCR |
| Nodal _R | GGTTGGTATCGTTTCAGCAGGC | qPCR |
| Sox2_F | TACAGCATGATGCAGGAGCAG | qPCR |
| Sox2_R | TCATGTAGGTCTGCGAGCTG | qPCR |
| Tbx6_F | CCTGAGCTTGGAGAACCAGG | qPCR |
| Tbx6_R | GGCCAGTGACTGATACTCGG | qPCR |
| gDNA_control_F | CCCCTTTCTGAAGCACTCTG | qPCR |
| gDNA_control_R | TAAGGCGTCATTTCCCAAAG | qPCR |

Development • Supplementary information

**Table S8. Antibodies used for Immunofluorescence and ChIP** References and details on antibody validation are listed on the manufacturer’s websites.

| Description | Catalog number | Company | Host Organism | Concentration | Application |
|---|---|---|---|---|---|
| α-T | #81694 | Cell Signaling Technology | Rabbit | 1:250 in PBSTB | IF |
| α-Sox2 | AF2018 | R&D | Goat | 1:250 in PBSTB | IF |
| α-Foxa2 | sc-6554 | Santa Cruz | Goat | 1:250 in PBSTB | IF |
| α-Olig2 | AF2418 | R&D | Goat | Rabbit | IF |
| α-Nkx2.2 | ab191077 | Abcam | Rabbit | Rabbit | IF |
| Alexa Fluor 488 α-Rabbit | ab150073 | Abcam | Donkey | 1:250 in PBSTB | IF |
| Alexa Fluor 647 α-Goat | ab150135 | Abcam | Donkey | 1:250 in PBSTB | IF |
| α-T | AF2085 | R&D | Goat | 2.5 µg reaction | ChIP |
| α-Foxa2 | C15410343 | Diagenode | Rabbit | 2.5 µg/reaction | ChIP |
| H3K4me1 | pAb-194-050 | Diagenode | Rabbit | 1.5 µg/reaction | ChIP |
| H3K4me3 | ab8580 | abcam | Rabbit | 1.5 µg/reaction | ChIP |
| H3K27ac | ab4729 | abcam | Rabbit | 1.5 µg/reaction | ChIP |
| H3K27me3 | C15410069 | Diagenode | Rabbit | 1.5 µg/reaction | ChIP |
| H3k9me2 | A90-0042 | Diagenode | Rabbit | 1.5 µg/reaction | ChIP |

**Table S9. RNA-Seq experiments**

| sample | embryos | cells | de-duplicated mapped reads |
|---|---|---|---|
| **NotoP Noto$^{mC}$+/T$^{Ve}$+/Foxa2$^{mT}$+ E8.5 9-11S** | 5 | 250 | 3343606 |
| **MP T$^{Ve}$_high/Noto$^{mC}$-/Foxa2$^{mT}$- 9-11S** | 5 | 24 | 1198064 |
| **NotoP Noto$^{mC}$+/T$^{Ve}$+/Foxa2$^{mT}$ _high E9.5 24-28S** | 5 | 250 | 4788324 |
| **MP T$^{Ve}$_high/Noto$^{mC}$-/Foxa2$^{mT}$ - 24-28S** | 5 | 250 | 6246472 |
| **Noto$^{mC}$+/T$^{Ve}$+/Foxa2$^{mT}$ _high 4-11S** | 8 | 73 | 1664032 |
| **Noto$^{mC}$+/T$^{Ve}$+/Foxa2$^{mT}$ _high 24-32 S** | 5 | 90 | 1581548 |

Development • Supplementary information
