# Supplementary material for: Genome-wide identification of notochord enhancers comprising the regulatory landscape of the brachyury locus in mouse
Source: Development. 2023 Nov 9;150(22):dev202111. doi: 10.1242/dev.202111 (PMC10651091; doi:10.1242/dev.202111)
Supplement: Supplementary file 1 [file develop-150-202111_review_history.pdf]

Genome-wide identification of notochord enhancers comprising the regulatory landscape of the brachyury locus in mouse

Dennis Schifferl, Manuela Scholze-Wittler, Alba Villaronga Luque, Milena Pustet, Lars Wittler, Jesse V. Veenvliet, Frederic Koch and Bernhard G Herrmann

DOI: 10.1242/dev.202111

Editor: James Briscoe

Review timeline

Original submission:	22 June 2023
Editorial decision:	14 July 2023
First revision received:	7 September 2023
Editorial decision:	29 September 2023
Second revision received:	16 October 2023
Accepted:	17 October 2023

Original submission

First decision letter

MS ID#: DEVELOP/2023/202111

MS TITLE: Genome-wide identification of notochord enhancers comprising the regulatory landscape of the Brachyury (T) locus in mouse

AUTHORS: Dennis Schifferl, Manuela Scholze-Wittler, Alba Villaronga Luque, Milena Pustet, Lars Wittler, Jesse V. Veenvliet, Frederic Koch, and Bernhard G Herrmann

I have now received all the referees' reports on the above manuscript, and have reached a decision. The referees' comments are appended below, or you can access them online: please go to BenchPressand click on the 'Manuscripts with Decisions' queue in the Author Area.

As you will see, the referees express considerable interest in your work, but have some significant criticisms and recommend a substantial revision of your manuscript before we can consider publication. The referees ask for a series of clarifications and revisions to the text and figures that will remove ambiguities and help readers follow the study. In addition, both Referee 1 and Referee 2 ask for more details about the origin of the notochord cells induced in your study in order to justify the conclusion 'Our data demonstrate the essential role of Foxa2 in switching T expressing cells from a NMP/mesodermal trajectory to the notochordal fate'. Their points seem to be important to address in order to support this conclusion.

If you are able to revise the manuscript along the lines suggested, which may involve further experiments, I will be happy receive a revised version of the manuscript. Your revised paper will be re-reviewed by one or more of the original referees, and acceptance of your manuscript will depend on your addressing satisfactorily the reviewers' major concerns. Please also note that Development will normally permit only one round of major revision. If it would be helpful, you are welcome to contact us to discuss your revision in greater detail. Please send us a point-by-point response indicating your plans for addressing the referee's comments, and we will look over this and provide further guidance.

© 2023. Published by The Company of Biologists under the terms of the Creative Commons Attribution License (https://creativecommons.org/licenses/by/4.0/).

Please attend to all of the reviewers' comments and ensure that you clearly highlight all changes made in the revised manuscript. Please avoid using 'Tracked changes' in Word files as these are lost in PDF conversion. I should be grateful if you would also provide a point-by-point response detailing how you have dealt with the points raised by the reviewers in the 'Response to Reviewers' box. If you do not agree with any of their criticisms or suggestions please explain clearly why this is so.

Reviewer 1

Advance summary and potential significance to field

This manuscript by Schifferl et al. focuses on the dissection of the enhancer landscape shaping the specification of the mouse notochord, an important embryonic structure that serves as a critical source of patterning signals. As notochord morphogenesis relies on the activity of a core set of transcription factors comprised of T (Brachyury), Foxa2 and Noto, the authors employed a triple T-Foxa2-Noto reporter mouse line and an in vitro mouse ES cell (mESC) differentiation system whereby Foxa2 expression is inducibly overexpressed in T-expressing cells that also carry a Noto fluorescent reporter to define a notochord-specific enhancer atlas following integration of gene expression T/Foxa2 genomic binding mapping, histone mark profiling and chromatin accessibility data obtained from ATAC- and ChiP-seq analysis of FACS-sorted embryonic and mESC-derived notochord progenitors. Based on these data, the authors went further into characterizing T locus-associated enhancers correlating with Notochord or mesoderm-inducing activity and using in vivo reporter assays and regulatory element deletion experiments they functionally pinpoint the enhancer elements (TNE/TNE2) orchestrating T-driven notochord progenitor maintenance and specification thus extending their previous work (Schifferl et al. 2021). Overall, this is an elegant study with well-executed experiments and high-quality data that reflect the conclusions of the authors providing an important insight into a key developmental process.

Comments for the author

My only question/suggestion is related to the in vitro Foxa2 overexpression mESC-based system: the authors obtain a significant fraction of T-Foxa2 co-expressing/Noto-negative cells following Dox treatment which is also included in the bulk ChiP-seq analysis (Fig. 1F, Suppl figure 2). T-Sox2 co-expression has been previously associated with anterior primitive streak/mesendoderm/nascent endoderm precursor identities, which are mutually exclusive to emerging T+SOX2+ NMP-liked cells in vitro (Burtscher & Lickert 2009 and Tsakiridis et al 2014).
Moreover, recent in vitro-based work has shown that the segregation of notochord and definitive endoderm fates from a common T+FOXA2+ entity as well as from other T+ progenitors (NMPs/lateral plate mesoderm) relies on time-dependent calibration of TGF/BMP/Nodal signaling activities (Robles Garcia et al. 2023 bioRXiv doi: https://doi.org/10.1101/2023.06.01.543323 and Rito et al. 2023 bioRxiv doi: ttps://doi.org/10.1101/2023.02.27.530267). In light of these data can the authors provide some more information about the nature and temporal/signaling dynamics of the emergence of the Noto-negative/Foxa2-overexpressing population vs their Noto-positive counterparts in their Dox-treated cultures? Does Foxa2 overexpression in their system directly divert T+Sox2+ cells toward an axial mesoderm/mesendoderm/NotoP fate or does this take place in non-NMP cells also present in the D3 cultures (as these do not usually consist of 100% T+Sox2+ cells)? Are the resulting Dox-treated Noto-negative cells nascent endoderm cells? How is Nodal signalling affected by Foxa2 overexpression? Inclusion of just a few additional time course data indicating the expression of cell fate (e.g. SOX2/SOX17) /signaling effector (e.g. SMAD2/3)-indicative markers in the cultures (by immunostaining/FACS analysis of + vs – Dox cells) would aid the detailed characterization of the system providing further valuable information on Notochord progenitor induction. This could be complemented with further analysis of the ChiP-seq data looking at the non-Notochord progenitor associated T/Foxa2 genomic targets that are marked by enhancer -associated histone marks and the TF binding motifs within them: are these enriched in endoderm-associated genes/transcriptional regulators and SMAD2/3 binding?

Reviewer 2

Advance summary and potential significance to field

© 2023. Published by The Company of Biologists under the terms of the Creative Commons Attribution License (https://creativecommons.org/licenses/by/4.0/).

In this paper the authors set out to characterise the regulatory genome of notochord, a critical signalling centre for developmental patterning. They do this by profiling chromatin accessibility of purified notochord cells from mouse embryos. They then develop an in vitro differentiation model using inducible TF overexpression to generate notochord cells from mouse embryonic stem cells differentiations. This system can generate larger cell numbers, which allows the authors to perform ChIP-seq for key notochord transcription factors T, Foxa2, and histone modifications associated with enhancers.

With this information at hand, the authors characterise the regulatory landscape of the T locus. They identify 10 open regions, one of them corresponding to their previously characterised TNE enhancer. Through reporter assays of three other regions and CRISPR deletion 5 regions, the authors focused a second enhancer termed TNE2.

In my opinion, the main advancement of this paper is the dissection of which elements are required for T expression, and/or are able drive reporter expression in the right tissue, combined with the information the authors have to the transcription factors and chromatin modifications bound to these elements.

Comments for the author

1. In the abstract the authors state “Our data demonstrate the essential role of Foxa2 in switching T expressing cells from a NMP/mesodermal trajectory to the notochordal fate.” What is evidence for this statement? Is this referring to the binding patters in Fig 2B? How does this statement fit with the previously-identified TNE element, critical for tail notochord development but not bound by Foxa2?

2. The paper would benefit a clearer explanation of why certain elements get chosen and the relevance of their TF binding profile.

The rationale for choosing specific elements for reporters or deletion experiments is not always clear. The current description includes some characteristics (some TFs that are binding, accessibility status or histone marks) for some of the elements, and then some of experiments done, but the logic is hard to follow. For example, end of page 7/beginning of 8 describes elements TE1, TE3, TE9, TE8 and TE6. This is followed by TE3 reporter and then TE7, which was outside the initial region that was being examined. During the description of the different elements binding of Sox2, beta-Catenin and Tbx6 are introduced, but it is not clear this information is used to choose which elements to pursue.

3. The previously identified TNE element, critical for notochord development (Schifferl et al., 2021) only shows binding of T and not Foxa2 in Figure 3. TE1 displays the same binding profiles, yet deletion of this element does not result in embryonic defects. Is there any other difference between these elements that could potentially explain the difference?

4. All deletion phenotypes apart from TNE2 refer to supplemental tables S4 S5, etc, I was only able to find Tables S1-3. What type of information about the phenotype is given in said tables? I realize it is negative data but it is an important part of the story.

5. Rationale for selection specific coordinates for enhancers. It does not seem to have been performed in any automated way (through peak calling or intersection of datasets). It is not obvious from the coverage in Figure 3 why TNE2 would extend so much to the right, or why TE9 would not be grouped with the left-hand-side of TNE2. An additional complication is the fact that if the 5kb scale bar is in Figure 3 is correct, the TNE2 box highlighted in grey and outlined in red cannot be 4.4 kb (the reported size for this element in the reporter assays in Fig S6A and the genotyping of deletion Fig S7).

6. The authors make the claim “The double KO phenotype shows that the combined activity of these two enhancers is required and sufficient for notochord formation and differentiation (…).” In order to claim these two enhancers (TNE and TNE2) are sufficient, one would have to show that they can drive notochord formation in the absence of any of the other regulatory elements. I

© 2023. Published by The Company of Biologists under the terms of the Creative Commons Attribution License (https://creativecommons.org/licenses/by/4.0/).

realize the authors have performed many deletions to rule out that many of the other elements on their own are required but it is quite difficult to show TNE and TNE2 are sufficient, in my opinion.

Additional minor comments:
- In page 7 the description to Figure 2E needs some clarification edits. It reads "Figure 2E lists the enhancers according to cat 1-7 found next to the 146 genes, showing examples of important well-known notochord marker genes" when the upset plot is depicting the genes, not the enhancers.
- In Figure S8, the labels for Olig2 and Nkx2.2 are switched.

Reviewer 3

Advance summary and potential significance to field

This is a very good paper that expands our understanding of gene regulation in the early notochord. The data is solid and will interest a broad range of researchers in this field. However, how the data is presented is confusing (see below). While this reviewer can figure out (I think) what the authors are concluding, this will likely not be the case for most people reading this paper. In particular, the paper would benefit from an expansion of the results/discussion (there really is not a discussion of the results in the current version). The authors have generated a large amount of data that I believe the field would look over very closely. While the authors were interested in one specific question, the paper would benefit from a broader analysis of their data (some suggestions below). These analyses would not require any additional bench experiments.

Comments for the author

General comment. The paper reads as a big run-on sentence in that there are three subsections for all the results/discussions. Makes it difficult to read. Suggest breaking the sections up into individual ideas/results.

Fig 4. It is very unclear what the reader should look at in the histology sections. While I am familiar with the morphology in this region, most readers of the article likely will not be. Please add anatomical labels and/or arrows.

Fig 4B-F Need more labels. Assume (and I really don't want to assume anything when reading an article in Development) that each of the top two images is from the upper crosssection? In B, showing the section distal from the tail, shouldn't there be T and noto? I zoomed as far as I could and I could not tell what the staining is (it is neither green nor pink). The 2X in A, C, E are useless. Need a ~50X zoom not a 2X zoom which would show the gaps in staining, which I think is the point of these images.

It is impossible to understand the paper without looking, in detail, at most of the supplemental figures, which means that these figures should not be supplemental. In particular, Sup fig 6 is essential for understanding the function of TNE enhancers, which are shown being KO in figure 4 of the paper. Without the Sup info shown in sup fig 6 there would be no reason to do the experiment shown in fig 4.

The paper needs a diagram showing where all the TNEs are relative to T. Figure 3 is fine to include, but a summary of the data is needed. For example, I am confused about what TNE refers to. Is it a deletion of multiple enhancers or just the one noted as TNE on the figure? I think they are two different regions, which would be clear from a diagram.

The paper needs a conclusion figure showing how the TNEs work together/aren't needed.

As a PI who has done work in this area, why was the triple Noto/FoxA2/T reported used? Noto by itself would have been sufficient as a reporter to purify the cells. I was hoping that maybe there was a subset of noto cells that did NOT express Foxa2/T in the node/caudal end, which would have been excluded by using the triple reporter (that would be really interesting). I am confused why all three reporters were used.

© 2023. Published by The Company of Biologists under the terms of the Creative Commons Attribution License (https://creativecommons.org/licenses/by/4.0/).

“Notochordal cells” have a very specific meaning in the literature. These are the cells in the annulus fibrosis that retain their “notochord” attributes, and many people think of this cell population as the disk stem cells.
That is not the cell population that the authors are referring to in this paper. A suggestion is that this term either not be used or clarified in the text (term used multiple times in the abstract and elswehere).

I have no idea what “maximum intensity” means. This term is used to describe every fluorescent image.

At least one paper has done a similar gene expression profiling analysis as the current report (see: https://www.ncbi.nlm.nih.gov/pmc/articles/PMC5585380/). How the current expression data set compares to published ones would be useful. The above paper was done at 12.5 (and P0), which is later than the analysis in the current paper. A comparison between gene expression over time in the notochord would be of immense interest to the field.

Minor:
Typo: “The data suggest that the activity of TE7 is increasing during notochord differentiation, which might explain that (think “that” should be “why”?) it was not detected in our differential ATAC-seq data derived from caudal end NotoPs.”

On this sentence: TNE2, on the other hand, is required for proper notochord cell specification and differentiation, since embryos lacking TNE show a dispersion of NotomC+ cells into all cell lineages of the tailbud, while the notochord is missing and the NotoP domain is shrinking during tail outgrowth (Schifferl et al., 2021).
- Should the start of the sentence be TNE (and not TNE2)?

First revision

Author response to reviewers' comments

Reviewer 1 Advance Summary and Potential Significance to Field:
This manuscript by Schifferl et al. focuses on the dissection of the enhancer landscape shaping the specification of the mouse notochord, an important embryonic structure that serves as a critical source of patterning signals. As notochord morphogenesis relies on the activity of a core set of transcription factors comprised of T (Brachyury), Foxa2 and Noto, the authors employed a triple T-Foxa2-Noto reporter mouse line and an in vitro mouse ES cell (mESC) differentiation system whereby Foxa2 expression is inducibly overexpressed in T- expressing cells that also carry a Noto fluorescent reporter to define a notochord-specific enhancer atlas following integration of gene expression, T/Foxa2 genomic binding mapping, histone mark profiling and chromatin accessibility data obtained from ATAC- and ChiP-seq analysis of FACS-sorted embryonic and mESC-derived notochord progenitors. Based on these data, the authors went further into characterizing T locus-associated enhancers correlating with Notochord or mesoderm-inducing activity and using in vivo reporter assays and regulatory element deletion experiments they functionally pinpoint the enhancer elements (TNE/TNE2) orchestrating T- driven notochord progenitor maintenance and specification thus extending their previous work (Schifferl et al. 2021). Overall, this is an elegant study with well-executed experiments and high-quality data that reflect the conclusions of the authors providing an important insight into a key developmental process.

RESPONSE: We thank the reviewer for the very positive assessment of our work.

Reviewer 1 Comments for the Author:
My only question/suggestion is related to the in vitro Foxa2 overexpression mESC-based system: the authors obtain a significant fraction of T-Foxa2 co- expressing/Noto-negative cells following Dox treatment which is also included in the bulk ChiP-seq analysis (Fig. 1F, Suppl figure 2). T-Sox2

© 2023. Published by The Company of Biologists under the terms of the Creative Commons Attribution License (https://creativecommons.org/licenses/by/4.0/).

co-expression has been previously associated with anterior primitive streak/mesendoderm/nascent endoderm precursor identities, which are mutually exclusive to emerging T+SOX2+ NMP-liked cells in vitro (Burtscher & Lickert 2009 and Tsakiridis et al 2014). Moreover, recent in vitro-based work has shown that the segregation of notochord and definitive endoderm fates from a common T+FOXA2+ entity as well as from other T+ progenitors (NMPs/lateral plate mesoderm) relies on time-dependent calibration of TGF/BMP/Nodal signaling activities (Robles Garcia et al. 2023
bioRXiv doi: https://doi.org/10.1101/2023.06.01.543323 and Rito et al. 2023 bioRxiv doi: ttps://doi.org/10.1101/2023.02.27.530267). In light of these data, can the authors provide some more information about the nature and temporal/signaling dynamics of the emergence of the Noto-negative/Foxa2- overexpressing population vs their Noto-positive counterparts in their Dox-treated cultures?

RESPONSE: We have analyzed the expression of marker genes in T/Foxa2-co-expressing Noto- CFP- and Noto-CFP+ cells. Both express Noto mRNA. On the other hand, Noto-CFP- cell numbers (P14) decrease from d3 to d4, whereas Noto-CFP+ cell numbers (P7) increase during differentiation. These data suggest that the reporter activity of the Noto-CFP- population is still too low to be detected by FACS sorting, but after some delay and reporter accumulation those cells will become CFP+ as well. CFP is the least sensitive reporter of the three used in our experiments. For details please see the revised Figure S2D.

Does Foxa2 overexpression in their system directly divert T+Sox2+ cells toward an axial mesoderm/mesendoderm/NotoP fate or does this take place in non-NMP cells also present in the D3 cultures (as these do not usually consist of 100% T+Sox2+ cells)?

RESPONSE: We do not think that NotoP cells derive from T+Sox2+ NMPs, but from a population of T+Sox2+ cells that can either form NMPs or NotoPs. It is known that a combination of WNT signaling (activating T) and (high) Nodal activity (inducing Foxa2) in the primitive streak is required for NotoP induction. Our system is designed such that from day2 on Foxa2 is upregulated as soon as T expression commences, in response to Chiron (WNT activation) and presumably prior to the NMP stage (day 3). Our data thus suggest the existence of a T+ stem cell-like cell that can give rise to either NMPs or NotoPs.

Are the resulting Dox-treated Noto-negative cells nascent endoderm cells?

RESPONSE: Please, see above. We have detected only a small cell group (P10; 2.1% of the total), which might represent nascent endoderm cells. These cells down-regulate T and Foax2 from d3 to d4 and at the same time up-regulate Sox17.

How is Nodal signalling affected by Foxa2 overexpression?

RESPONSE: Nodal is down-regulated at first on day 3, but reverts to its initial expression level in NotoP-diff cells (P7) at day 4 relative to T- cells on day 3.

Inclusion of just a few additional time course data indicating the expression of cell fate (e.g. SOX2/SOX17) /signaling effector (e.g. SMAD2/3)- indicative markers in the cultures (by immunostaining/FACS analysis of + vs – Dox cells) would aid the detailed characterization of the system providing further valuable information on Notochord progenitor induction.

RESPONSE: We have re-done the experiment now showing qPCR data for days 3, 3.25 and 4 for 7 important TFs and Nodal, providing a much deeper characterization of our in vitro model for generating NotoPs in culture.

This could be complemented with further analysis of the ChiP-seq data looking at the non-Notochord progenitor associated T/Foxa2 genomic targets that are marked by enhancer - associated histone marks and the TF binding motifs within them: are these enriched in endoderm-associated genes/transcriptional regulators and SMAD2/3 binding?

RESPONSE: This question of the reviewer relates to the notion expressed further above that T/Foxa2 targets not related to notochord might represent endoderm-specific enhancers.

© 2023. Published by The Company of Biologists under the terms of the Creative Commons Attribution License (https://creativecommons.org/licenses/by/4.0/).

However, our data suggest that the reporter T+/Foxa2+/Noto- cells are bound to become Noto reporter positive, rather than representing endoderm-like cells. Since Foxa2 is a proven and T a putative “pioneer” TF, we expect all targets of both factors bound in the differentiated cells at day 4. Most of the Foxa2 bound sites presumably would be related to endoderm-associated genes. However, without endoderm-specific in vivo ATAC and gene expression data and without in vitro enhancer chromatin signature data of purified endoderm cells, we would not be able to identify true endoderm gene-associated (active) enhancers and analyze them further.

Reviewer 2 Advance Summary and Potential Significance to Field:
In this paper the authors set out to characterise the regulatory genome of notochord, a critical signaling center for developmental patterning. They do this by profiling chromatin accessibility of purified notochord cells from mouse embryos. They then develop an in vitro differentiation model using inducible TF overexpression to generate notochord cells from mouse embryonic stem cells differentiations. This system can generate larger cell numbers, which allows the authors to perform ChIP-seq for key notochord transcription factors T, Foxa2, and histone modifications associated with enhancers.
With this information at hand, the authors characterize the regulatory landscape of the T locus. They identify 10 open regions, one of them corresponding to their previously characterized TNE enhancer. Through reporter assays of three other regions and CRISPR deletion 5 regions, the authors focused a second enhancer, termed TNE2.

In my opinion, the main advancement of this paper is the dissection of which elements are required for T expression, and/or are able drive reporter expression in the right tissue, combined with the information the authors have to the transcription factors and chromatin modifications bound to these elements.

Reviewer 2 Comments for the Author:
1. In the abstract the authors state “Our data demonstrate the essential role of Foxa2 in switching T expressing cells from a NMP/mesodermal trajectory to the notochordal fate.” What is evidence for this statement? Is this referring to the binding patters in Fig 2B? How does this statement fit with the previously- identified TNE element, critical for tail notochord development but not bound by
Foxa2?

RESPONSE: The statement was referring to the fact that co-expression of Foxa2 with T from day3 to 4 generates a considerable fraction of Noto+ cells, while T alone under the same conditions (Chir treatment) generates mesoderm. However, we agree that the sentence cited by the reviewer may be misleading, and therefore changed it in the revised manuscript.
With respect to the notochord enhancer TNE: Foxa2 obviously is not co-binding with T on all notochord enhancers, but on the T locus it is co-binding with T on TNE2, and TNE and TNE2 synergize. Without Foxa2, however, notochord would not (or very scarcely) be made.

2. The paper would benefit a clearer explanation of why certain elements get chosen and the relevance of their TF binding profile.

The rationale for choosing specific elements for reporters or deletion experiments is not always clear. The current description includes some characteristics (some TFs that are binding, accessibility status or histone marks) for some of the elements, and then some of experiments done, but the logic is hard to follow. For example, end of page 7/beginning of 8 describes elements TE1, TE3, TE9, TE8 and TE6. This is followed by TE3 reporter and then TE7, which was outside the initial region that was being examined. During the description of the different elements, binding of Sox2, beta-Catenin and Tbx6 are introduced, but it is not clear this information is used to choose which elements to pursue.

RESPONSE: We would like to point out that we examined all putative enhancer elements (6 out of 8: TE1, TNE, TE3, TE9, TNE2, TE7) identified by ATAC in NotoPs in vivo and bound by T in NotoP-diff in vitro, by knockout analysis in embryos. In total we examined 4 out of the 6 elements also by reporter assays in embryos (TE1 and TE9 were not done since they did not show an essential role upon LoF).
The binding of Sox2, beta-Catenin and Tbx6 provide extra information, which may be useful to the

© 2023. Published by The Company of Biologists under the terms of the Creative Commons Attribution License (https://creativecommons.org/licenses/by/4.0/).

community.

3. The previously identified TNE element, critical for notochord development (Schifferl et al., 2021) only shows binding of T and not Foxa2 in Figure 3. TE1 displays the same binding profiles, yet deletion of this element does not result in embryonic defects. Is there any other difference between these elements that could potentially explain the difference?

RESPONSE: The only difference we have observed is the fact that TNE, but not TE1 binds b- Catenin. Besides this observation there is no indication why TNE is an essential notochord enhancer and TE1 is not.

4. All deletion phenotypes apart from TNE2 refer to supplemental tables S4, S5, etc, I was only able to find Tables S1-3. What type of information about the phenotype is given in said tables? I realize it is negative data but it is an important part of the story.

RESPONSE: Supplemental tables 4-8 are included in the supplementary data file. Table S4 lists the ES cell lines (reporter and mutants), table S5 the phenotype examination of the enhancer knockout lines, which showed no essential function of TE1, TE3, TE7 and TE9, included in this study.

5. Rationale for selection specific coordinates for enhancers. It does not seem to have been performed in any automated way (through peak calling or intersection of datasets). It is not obvious from the coverage in Figure 3 why TNE2 would extend so much to the right, or why TE9 would not be grouped with the left-hand-side of TNE2. An additional complication is the fact that if the 5kb scale bar is in Figure 3 is correct, the TNE2 box highlighted in grey and outlined in red cannot be 4.4 kb (the reported size for this element in the reporter assays in Fig S6A and the genotyping of deletion Fig S7).

RESPONSE: The rationale for selection of the putative enhancer region of TNE2 is mainly based on the co-binding of T and Foxa2 at 3 neighboring sites. TE9, on the other hand binds T and Sox2, but not Foxa2. This distinguishes the 2 elements. For TE3, three overlapping (sub)regions have been analyzed separately (see Suppl. Material). Indeed, the elements chosen for analysis were mainly guided by peak sizes and TF consensus sites contained in them, rather than in an automated way.
The scale bar 5kb on the bottom of Figure 3 is indeed wrong. We thank the reviewer for pointing this out. We have corrected the scale bar in the revised manuscript.

6. The authors make the claim “The double KO phenotype shows that the combined activity of these two enhancers is required and sufficient for notochord formation and differentiation (…).” In order to claim these two enhancers (TNE and TNE2) are sufficient, one would have to show that they can drive notochord formation in the absence of any of the other regulatory elements. I realize the authors have performed many deletions to rule out that many of the other elements on their own are required but it is quite difficult to show TNE and TNE2 are sufficient, in my opinion.

RESPONSE: We agree with the reviewer that the term “sufficient” is incorrect and accordingly deleted it in the revised manuscript.

Additional minor comments:
-In page 7 the description to Figure 2E needs some clarification edits. It reads “Figure 2E lists the enhancers according to cat 1-7 found next to the 146 genes, showing examples of important well-known notochord marker genes” when the upset plot is depicting the genes, not the enhancers.

RESPONSE: This sentence has been corrected in the revised manuscript.

-In Figure S8, the labels for Olig2 and Nkx2.2 are switched.

RESPONSE: We thank the reviewer for pointing out this mistake to us. We have corrected the captions accordingly.

© 2023. Published by The Company of Biologists under the terms of the Creative Commons Attribution License (https://creativecommons.org/licenses/by/4.0/).

Reviewer 3 Advance Summary and Potential Significance to Field:
This is a very good paper that expands our understanding of gene regulation in the early notochord. The data is solid and will interest a broad range of researchers in this field. However, how the data is presented is confusing (see below). While this reviewer can figure out (I think) what the authors are concluding, this will likely not be the case for most people reading this paper. In particular, the paper would benefit from an expansion of the results/discussion (there really is not a discussion of the results in the current version). The authors have generated a large amount of data that I believe the field would look over very closely. While the authors were interested in one specific question, the paper would benefit from a broader analysis of their data (some suggestions below). These analyses would not require any additional bench experiments.

Reviewer 3 Comments for the Author:
General comment. The paper reads as a big run-on sentence in that there are three subsections for all the results/discussions. Makes it difficult to read. Suggest breaking the sections up into individual ideas/results.

RESPONSE: We apologize for the inconvenience put to the reviewer. However, we respectfully disagree and beleive that the text flows well and arguments are easy to follow.

Fig 4. It is very unclear what the reader should look at in the histology sections. While I am familiar with the morphology in this region, most readers of the article likely will not be. Please add anatomical labels and/or arrows.

RESPONSE: We have included labels for the neural tube, which shows the most dramatic anatomical abnormality and most impressively reflects the consequences of an absent notochord.

Fig 4B-F Need more labels. Assume (and I really don't want to assume anything when reading an article in Development) that each of the top two images is from the upper crosssection? In B, showing the section distal from the tail, shouldn't there be T and noto? I zoomed as far as I could and I could not tell what the staining is (it is neither green nor pink). The 2X in A, C, E are useless. Need a ~50X zoom not a 2X zoom, which would show the gaps in staining, which I think is the point of these images.

RESPONSE: We have improved the labeling in the images, and showed an additional enlargement of the notochord section in Fig. 4D to make single and double positive cells better visible. The "2X" in A',E',I' indicates 2X higher intensity (not enlargement) to emphasize the lower T expression level in the mutant. We think the disruptions in the notochord are well visible on the Noto-mC staining image.

It is impossible to understand the paper without looking, in detail, at most of the supplemental figures, which means that these figures should not be supplemental. In particular, Sup fig 6 is essential for understanding the function of TNE enhancers, which are shown being KO in figure 4 of the paper. Without the Sup info shown in sup fig 6 there would be no reason to do the experiment shown in fig 4.

RESPONSE: We agree with the reviewer that the TNE2 reporter data belong to the main figures. Therefore, we have moved the TNE2 reporter assay from Fig. S6 to main figure 3. Since the knockout of TE3 and TE7 did not show an essential function of these elements we consider the reporter assays for these elements as supplementary information.

The paper needs a diagram showing where all the TNEs are relative to T. Figure 3 is fine to include, but a summary of the data is needed. For example, I am confused about what TNE refers to. Is it a deletion of multiple enhancers or just the one noted as TNE on the figure? I think they are two different regions, which would be clear from a diagram.
RESPONSE: We have included a schematic drawing at the bottom of figure 3A. TNE has been published recently (Schifferl et al. 2021); it is the single enhancer noted as TNE on the figure. TNE2 comprises 3 ATAC peaks bound by T and Foxa2, as stated in the text. All other T locus putative enhancers are abbreviated TE, and indicated by grey boxes.

© 2023. Published by The Company of Biologists under the terms of the Creative Commons Attribution License (https://creativecommons.org/licenses/by/4.0/).

The paper needs a conclusion figure showing how the TNEs work together/aren't needed.

RESPONSE: We have included available information on the conclusion figure (Fig. 5) as requested by the reviewer.

As a PI who has done work in this area, why was the triple Noto/FoxA2/T reported used? Noto by itself would have been sufficient as a reporter to purify the cells. I was hoping that maybe there was a subset of noto cells that did NOT express Foxa2/T in the node/caudal end, which would have been excluded by using the triple reporter (that would be really interesting). I am confused why all three reporters were used.

RESPONSE: The main reason for the triple reporter line is that it clearly identifies the desired notochord cells, and allows to distinguish notochord from mesoderm and from endoderm. Noto alone would be sufficient to isolate notochord cells, but not mesoderm. For distinguishing notochord from mesoderm enhancers, we needed to isolate both cell types. T is expressed in both, notochord and mesoderm, Foxa2 in notochord and endoderm. Thus, Foxa2 is used to distinguish notochord and endoderm from mesoderm.

"Notochordal cells" have a very specific meaning in the literature. These are the cells in the annulus fibrosis that retain their "notochord" attributes, and many people think of this cell population as the disk stem cells. That is not the cell population that the authors are referring to in this paper. A suggestion is that this term either not be used or clarified in the text (term used multiple times in the abstract and elswehere).

RESPONSE: We found that the term "notochordal" is, even in recent literature, not strictly used only for cells in the annulus fibrosis as suggested by the reviewer. Still, we followed the recommendation of the reviewer and abstained from using the term "notochordal" as much as possible in the revised manuscript.

I have no idea what "maximum intensity" means. This term is used to describe every fluorescent image.

RESPONSE: We have given more details and a reference about the imaging tool "maximum intensity projection" in the methods section.

At least one paper has done a similar gene expression profiling analysis as the current report (see: https://www.ncbi.nlm.nih.gov/pmc/articles/PMC5585380/). How the current expression data set compares to published ones would be useful. The above paper was done at 12.5 (and P0), which is later than the analysis in the current paper. A comparison between gene expression over time in the notochord would be of immense interest to the field.

RESPONSE: Indeed, a comparison of gene expression over time of development would be of high interest. However, the datasets need to be compatible, and this is not the case between ours and the one cited by the reviewer (different RNA-seq library kits used). E.g., we used UMIs and a ligation-based method, whereas the published data were generated differently resulting in different outcomes with respect to quantification of expression levels. In addition, different reporters were used in the other study.

Minor:
Typo: "The data suggest that the activity of TE7 is increasing during notochord differentiation, which might explain that (think "that" should be "why"?) it was not detected in our differential ATAC-seq data derived from caudal end NotoPs."

RESPONSE: We agree that "why" is better in this context and corrected the manuscript accordingly.

On this sentence: TNE2, on the other hand, is required for proper notochord cell specification and differentiation, since embryos lacking TNE show a dispersion of NotomC+ cells into all cell lineages of the tailbud, while the notochord is missing and the NotoP domain is shrinking during tail outgrowth (Schifferl et al., 2021).

© 2023. Published by The Company of Biologists under the terms of the Creative Commons Attribution License (https://creativecommons.org/licenses/by/4.0/).

Should the start of the sentence be TNE (and not TNE٢)?

RESPONSE: This sentence indeed lacked clarity and was formulated anew.

Second decision letter

MS ID#: DEVELOP/2023/202111

MS TITLE: Genome-wide identification of notochord enhancers comprising the regulatory landscape of the Brachyury (T) locus in mouse

AUTHORS: Dennis Schifferl, Manuela Scholze-Wittler, Alba Villaronga Luque, Milena Pustet, Lars Wittler, Jesse V. Veenvliet, Frederic Koch, and Bernhard G Herrmann

I have now received all the referees reports on the above manuscript, and have reached a decision. The referees' comments are appended below, or you can access them online: please go to BenchPress and click on the 'Manuscripts with Decisions' queue in the Author Area.

The overall evaluation is positive and we would like to publish a revised manuscript in Development, provided that the referees' comments can be satisfactorily addressed. Please provide a clearer explanation of the image processing, as requested by Reviewer 3. I also agree with Reviewer 3 that the study would be strengthened by including comparisons with related data such as the study by Peck et al. (PMID: 28874804).Please attend to all of the reviewers' comments in your revised manuscript and detail them in your point-by-point response. If you do not agree with any of their criticisms or suggestions explain clearly why this is so. If it would be helpful, you are welcome to contact us to discuss your revision in greater detail. Please send us a point-by-point response indicating your plans for addressing the referees’ comments, and we will look over this and provide further guidance.

Reviewer 1

Advance summary and potential significance to field

I am happy with the changes/response of the authors to my comments and I support the manuscript's publication.

Comments for the author

N/A

Reviewer 2

Advance summary and potential significance to field

I thank the authors for their efforts to make the manuscript easier to follow particularly with the additional diagrams in the last figure.

This is a timely and important piece of work, as well as a useful resource for the field that should be published.

Comments for the author

I would suggest clarifications in two parts of the manuscript, which can be discussed with the editorial team directly.

1) The explanation of Figure 2E in the main text is not clear, even for someone who regularly produces plots like the one shown. If I understand correctly the authors have identifies the candidate target genes for elements of different categories and are plotting how many genes have

© 2023. Published by The Company of Biologists under the terms of the Creative Commons Attribution License (https://creativecommons.org/licenses/by/4.0/).

elements belonging to the different categories. The figure does not exactly “lists these genes and the enhancers according to cat 1-7 found in their neighborhood”.

2) I find the discussion on where the NotoP cells come from very helpful. However the sentence in page 10 “The procedure effects Foxa2 expression in dependance on T induction by WNT, that is as soon as T expression commences.” is, in my opinion very confusing.

Reviewer 3

Advance summary and potential significance to field

see original review

Comments for the author

Comments:
- 2X enhanced in fig 4 needs to be explained much better. How was this “enhanced”? This is a general comment, which is also relevant to fig 4, “enhanced” can mean many different things along with “maximum intensity.”
- I appreciate the addition of the Wallis (1989) reference explaining maximum intensity. Still, that paper did not deal with confocal images and was published in an age where images could not easily be edited on a computer.
- Since large parts of the paper are based on fluorescent image intensity, it is essential that the method used to quantify the fluorescent intensity is extensively referenced/explained.

A single label was added to fig 4. While this helps, there are many other places where it is very unclear what the reader should be looking at (see fig 3B for example, which was added in the revision).

Second revision

Author response to reviewers' comments

Response to Reviewers’ Comments:

Editor
The overall evaluation is positive and we would like to publish a revised manuscript in Development, provided that the referees' comments can be satisfactorily addressed. Please provide a clearer explanation of the image processing, as requested by Reviewer 3. I also agree with Reviewer 3 that the study would be strengthened by including comparisons with related data such as the study by Peck et al. (PMID: 28874804).

RESPONSE: We have performed a comparison of our dataset with three datasets published previously and provide a Venn diagram (Fig. S2C) showing the overlap and an Excel table (Table S2) listing the genes expressed in single datasets and their overlaps.

Reviewer 2 Comments for the Author:
I would suggest clarifications in two parts of the manuscript, which can be discussed with the editorial team directly.

1) The explanation of Figure 2E in the main text is not clear, even for someone who regularly produces plots like the one shown. If I understand correctly the authors have identifies the candidate target genes for elements of different categories and are plotting how many genes have elements belonging to the different categories. The figure does not exactly “lists these genes and the enhancers according to cat 1-7 found in their neighborhood”.

© 2023. Published by The Company of Biologists under the terms of the Creative Commons Attribution License (https://creativecommons.org/licenses/by/4.0/).

RESPONSE: We have changed the text criticized by the reviewer and hope it is better conceivable now.

2) I find the discussion on where the NotoP cells come from very helpful. However, the sentence in page 10 “The procedure effects Foxa2 expression in dependance on T induction by WNT, that is as soon as T expression commences.” is, in my opinion, very confusing.

RESPONSE: We have re-formulated the sentence criticized by the reviewer in the revised manuscript.

***** Reviewer 3 Advance Summary and Potential Significance to Field:
see original review

Reviewer 3 Comments for the Author:
Comments:
-2X enhanced in fig 4 needs to be explained much better. How was this “enhanced”?

RESPONSE: We thank the reviewer for pointing out that the term „enhanced“ can mean different operations and is misleading in this context. To avoid confusion, we have removed the term “enhanced” from the plates and explained in the legend that “maximum brightness was equally adjusted in the insets”. We added to the method section that he difference between maximum brightness (white level) and minimum brightness (black level) was reduced by 50% to display weaker signals in notochord cells. We hope that the procedure for imaging has now been clarified satisfactorily.

This is a general comment, which is also relevant to fig 4, “enhanced” can mean many different things along with “maximum intensity.”
-I appreciate the addition of the Wallis (1989) reference explaining maximum intensity. Still, that paper did not deal with confocal images and was published in an age where images could not easily be edited on a computer.
-Since large parts of the paper are based on fluorescent image intensity, it is essential that the method used to quantify the fluorescent intensity is extensively referenced/explained.

RESPONSE: Unfortunately, to our knowledge Wallis et al is the only reference available on the subject. Even though the Wallis et al paper does not deal with micrographs acquired by fluorescent microscopy, the method for image rendering remains identical: For every pixel (X,Y) in a 3D image stack, the brightest value along the Z axis is displayed in the according position (X,Y) on a 2D plane. We explain this imaging procedure in the methods section.
In ZEN, as well as other widely used imaging software, maximum intensity projections can be obtained from 3D data without any specifications. We consulted Zeiss and were assured that ZEN software does not use any additional algorithms. Therefore, we think that providing information about the software version is sufficient to explain the processing step. We agree with the reviewer that maximum intensity projections would in fact not be an ideal method for exact quantitative measurements of fluorescence intensity. However, in Figure 3 and 4, our focus is to describe morphological features (such as the presence and distribution of notochord cells).

A single label was added to fig 4. While this helps, there are many other places where it is very unclear what the reader should be looking at (see fig 3B for example, which was added in the revision).

RESPONSE: We have labeled additional anatomical structures on the figure.

<u>Third decision letter</u>

MS ID#: DEVELOP/2023/202111

© 2023. Published by The Company of Biologists under the terms of the Creative Commons Attribution License (https://creativecommons.org/licenses/by/4.0/).

MS TITLE: Genome-wide identification of notochord enhancers comprising the regulatory landscape of the Brachyury (T) locus in mouse

AUTHORS: Dennis Schifferl, Manuela Scholze-Wittler, Alba Villaronga Luque, Milena Pustet, Lars Wittler, Jesse V. Veenvliet, Frederic Koch, and Bernhard G Herrmann
ARTICLE TYPE: Research Article

I am happy to tell you that your manuscript has been accepted for publication in Development, pending our standard ethics checks.

© 2023. Published by The Company of Biologists under the terms of the Creative Commons Attribution License (https://creativecommons.org/licenses/by/4.0/).
